# Effects of Co-Cultivation on the Phenolic Composition and Bioactive Properties of Basidiomycete Mycelia: Antioxidant, Photoprotective, Anti-Tyrosinase, and In Silico Evidence

**DOI:** 10.3390/molecules31152569

**Published:** 2026-07-23

**Authors:** Katielle Vieira Avelino, Maria Aparecida Pereira Garcez, Roberta Fernanda Rogonni Ferrari Giansante, Marisangela Isabel Wietzikoski Halabura, Caroline Domingues, Maria Graciela Iecher Faria Nunes, Nelson Barros Colauto, Lidiane Nunes Barbosa, Zilda Cristiani Gazim, Daniela Dib Gonçalves, Flávio Augusto Vicente Seixas, Antonio Laverde Junior, Juliana Silveira do Valle

**Affiliations:** 1 Graduate Program in Biotechnology Applied to Agriculture, Universidade Paranaense, Umuarama 87502-210, Brazil; katielle.avelino@edu.unipar.br (K.V.A.); maria181071@edu.unipar.br (M.A.P.G.); marisangela.halabura@edu.unipar.br (M.I.W.H.); gracielaiecher@prof.unipar.br (M.G.I.F.N.); cristianigazim@prof.unipar.br (Z.C.G.); 2 Graduate Program in Animal Science with Emphasis on Bioactive Products, Universidade Paranaense, Umuarama 87502-210, Brazil; roberta20588@edu.unipar.br (R.F.R.F.G.); lidianebarbosa@prof.unipar.br (L.N.B.); danieladib@prof.unipar.br (D.D.G.); 3 Graduate Program in Medicinal and Phytotherapeutic Plants in Primary Care, Universidade Paranaense, Umuarama 87502-210, Brazil; c.domingues@edu.unipar.br; 4 Graduate Program in Bioscience Technologies, Universidade Tecnológica Federal do Paraná, Toledo 85902-490, Brazil; nelsonbcolauto@gmail.com; 5 Department of Technology, Universidade Estadual de Maringá, Umuarama 87506-370, Brazil; favseixas@uem.br; 6 Laboratório de Química Orgânica e Biomateriais, Departamento de Química, Universidade Tecnológica Federal do Paraná, Londrina 86036-700, Brazil; aljunior@utfpr.edu.br

**Keywords:** fungal interactions, secondary metabolites, ultraviolet protection, melanogenesis, oxidative stress, molecular target prediction, GO enrichment, KEGG pathways, pharmacokinetic prediction, fungal biotechnology

## Abstract

Basidiomycetes produce a wide range of bioactive metabolites, yet the effects of fungal co-cultivation on phenolic composition and associated biological properties remain poorly understood. This study investigated the phenolic composition and antioxidant, photoprotective, and anti-tyrosinase activities of mycelial extracts obtained from four basidiomycetes cultivated either alone or in paired cultures. Phenolic compounds were characterized by HPLC, and the identified metabolites were further evaluated using target prediction, enrichment analyses, PASS prediction, ADME profiling, and toxicity assessment. Nine phenolic compounds were identified across ten cultivation systems. Catechin and syringic acid were detected only in selected paired cultures, indicating that fungal interactions altered phenolic composition. The highest antioxidant activities were observed for *P. sanguineus* and *T. polyzona* cultivated alone, while SPF reached 39 in *P. ostreatus*, *T. polyzona*, *Lentinus* + *Pycnoporus*, and *Pycnoporus* + *Trametes*. Tyrosinase inhibition reached 92.7% in the axenic culture of *P. ostreatus* and remained above 90% in selected co-cultures. Computational analyses identified enrichment of biological processes related to oxidative stress and UV responses, as well as pathways including PI3K-Akt, FoxO, HIF-1, and p53. PASS prediction indicated that catechin, quercetin, and myricetin showed the highest probabilities for antioxidant activity, inhibition of lipid peroxidation and NADPH oxidase, and melanin synthesis inhibition, while ADMET predicted good solubility and high gastrointestinal absorption for most of the identified metabolites. Fungal interactions altered the phenolic composition of basidiomycete mycelia and influenced their biological activities, indicating that co-cultivation may be a useful strategy for modulating the production of bioactive metabolites.

## 1. Introduction

Fungal metabolism can change in response to environmental influences, such as variations in pH and temperature, low nutrient availability, lighting conditions [1], and competition among species [2,3]. Competition is the most common interaction among white-rot fungi in the environment, occurring through competition for territory and nutrients, which can alter fungal physiology and metabolic activity. These environmental and biotic factors can influence fungal physiology and regulate the synthesis of secondary metabolites, including phenolic compounds and other bioactive molecules with ecological and biotechnological relevance [4].

In an attempt to gain control over substrates shared with other species, mycelial interactions can promote changes in mycelial morphology and trigger physiological responses that improve competitive fitness [5]. Changes can include regulation of oxidative stress and the production of secondary metabolites involved in the defensive response during competitive interactions [6]. Fungal co-cultivation has been recognized as a strategy to activate metabolic pathways that are poorly expressed under axenic conditions, including signal molecules, growth inhibitors, toxins, and by-products [7]. As a result, co-culture systems emerge as an approach for discovering new bioactive compounds and enhancing metabolite synthesis with potential pharmaceutical, nutraceutical, and cosmetic applications [8].

Basidiomycete fungi are known to frequently produce phenolic compounds as secondary metabolites, which can be detected in fruiting bodies [9], mycelium [10], and in the cultivation medium or substrate [11]. Phenolic acids and flavonoids, which exhibit antioxidant, anti-inflammatory, and antimicrobial activities [12,13], have also been linked to photoprotective effects and modulation of melanogenesis-related pathways, including tyrosinase inhibition [14,15,16]. However, the diversity and concentration of phenolics vary with fungal species and strain, cultivation conditions, and substrate composition [17,18]. Due to these properties, fungal extracts rich in phenolic compounds have potential for industrial applications, highlighting the importance of understanding the factors that influence their production.

In mushroom-producing fungi, the vegetative mycelium and the fruiting body may differ in their metabolic profiles and chemical composition [19,20]. Although studies on fruiting bodies predominate in the literature, the production of vegetative mycelium as a source of bioactive compounds can be advantageous when compared with fruiting bodies [21]. Mycelium can be produced in less time and in large quantities through fermentative bioprocesses in bioreactors. Cultivating fungi as vegetative mycelium in bioprocesses allows tighter control of temperature, oxygenation, culture medium composition, and other cultivation parameters, as well as improved control over contamination, thereby optimizing the production of bioactive compounds [22]. Such production strategies exhibit lower variability, are reproducible, and enable simple recovery of mycelial biomass, contributing to process scalability [23]. These characteristics make mycelial cultivation a suitable platform for investigating factors that modulate the production of fungal bioactive compounds.

Interactions among basidiomycetes can affect secondary metabolite production and, depending on the species involved, can lead to increases, decreases, or the emergence of compounds absent in monocultures [24,25,26]. For phenolic compounds, these responses are often expressed as changes in the occurrence and relative abundance of individual compounds rather than as increases in total phenolic content [27,28]. Such qualitative changes may alter biological properties even when total phenolic levels remain unchanged. Although phenolic content and antioxidant activity are frequently evaluated in co-culture systems, the specific phenolic compounds associated with these responses are rarely characterized. As a result, little is known about how interaction-induced changes in phenolic composition affect biological properties. This gap is particularly evident in basidiomycete co-cultures, where studies that combine phenolic characterization with antioxidant, photoprotective, and anti-tyrosinase evaluations remain scarce, limiting understanding of how changes in phenolic composition relate to biological activity.

The white-rot basidiomycetes *Lentinus crinitus*, *Pleurotus ostreatus*, *Pycnoporus sanguineus*, and *Trametes polyzona* differ in their reported metabolite profiles and biological activities [10,29,30,31]. Their taxonomic diversity, metabolic distinctiveness, and biotechnological relevance make them suitable models for investigating how interspecific interactions influence phenolic composition and associated biological properties.

This study presents an analysis of how fungal interactions affect metabolite composition and bioactive potential, integrating phenolic profiling with biological and in silico analyses in basidiomycete co-cultures.

Therefore, this study aimed to evaluate changes in phenolic composition induced by co-cultivation of four white-rot basidiomycetes and to investigate their potential relationship with antioxidant, photoprotective, and anti-tyrosinase activities. In silico analyses were used to explore the biological relevance of the identified phenolic compounds.

## 2. Results

### 2.1. Total Phenolic Content and Phenolic Profile of Axenic and Co-Culture Extracts

The phenolic composition of the mycelial extracts varied substantially among axenic and co-culture systems (Table 1). In general, monocultures exhibited higher total phenolic contents than paired cultures, especially *P. sanguineus* and *T. polyzona*, which showed the highest concentrations among the evaluated systems.

The chromatographic analysis revealed differences in the diversity and concentration of phenolic compounds of the mycelial biomasses (Table 1). Five phenolic acids, three flavonoids, and one phenolic aldehyde were identified in the mycelial extracts. The flavonoid myricetin was detected exclusively in the monoculture of *L. crinitus*, while catechin occurred exclusively in the mycelium extracts of some paired cultures. On the other hand, quercetin was detected in all mycelial extracts except in the *P. ostreatus* monoculture and *Pleurotus* + *Pycnoporus* co-culture. Gallic acid was present in all extracts, with the highest concentration detected in the *Pleurotus* + *Trametes* co-culture (41 µg g^−1^). This value was 1.5- and 2.5-fold higher than those observed in the respective monocultures.

Among the strains cultivated alone (Table 1), *L. crinitus* showed the highest diversity of phenolic compounds (7 compounds); however, it had the lowest total phenol content (53.5 µg g^−1^). *Pycnoporus sanguineus* mycelial biomass exhibited the lowest diversity of detected compounds (three compounds) but the highest content of phenols (111.7 µg g^−1^), mainly protocatechuic acid (41.8 µg g^−1^) and quercetin (52.1 µg g^−1^).

Among the paired cultures, the combinations *Lentinus* + *Pleurotus* and *Pycnoporus* + *Trametes* contained the greatest compound diversity (seven). Furthermore, *Pycnoporus* + *Trametes* had the highest total phenol content (96.2 µg g^−1^), while *Pleurotus* + *Trametes* had the second-highest phenol content (74.5 µg g^−1^). The paired cultures containing *Pleurotus* showed lower phenolic content than the respective monocultures. In contrast, the monoculture of *P. ostreatus* was among the cultures with the fewest phenolic compounds identified (four) but had the second-highest (*p* ≤ 0.05) total phenol content (97.4 µg g^−1^), with benzoic acid (60.5 µg g^−1^) as the primary compound.

The flavonoid catechin and the phenolic acid syringic acid were detected only in paired fungal cultures, indicating that fungal interactions can induce their production. Co-cultivation of *Lentinus* + *Pleurotus* showed significant concentrations of catechin (13.2 µg g^−1^) and syringic acid (4.9 µg g^−1^). When the mycelial biomass of *P. ostreatus* and *P. sanguineus* was cultivated alone, no *p*-coumaric acid was detected. In contrast, the production of *p*-coumaric acid was induced during the co-cultivation of the two species. However, the highest (*p* ≤ 0.05) concentration of p-coumaric acid was observed in the co-cultivation of *Lentinus* + *Pycnoporus* (5.9 µg g^−1^).

### 2.2. Antioxidant Activity of Axenic and Co-Culture Extracts

The antioxidant activity of the axenic and co-culture extracts varied according to the fungal combination and assay employed (Table 2). In general, monocultures exhibited stronger antioxidant activity than paired cultures, with particularly high antioxidant activity observed for *P. sanguineus* and *T. polyzona*, which showed the highest reducing power, DPPH scavenging activity, and FRAP values.

In general, paired cultures showed lower reducing power than the respective monocultures (Table 2). Among the evaluated systems, the *T. polyzona* monoculture exhibited the highest reducing power, followed by *P. sanguineus*.

None of the extracts achieved the DPPH radical-scavenging capacity of quercetin, the positive control. Among the fungal extracts, the monoculture of *T. polyzona* showed the highest scavenging activity (Table 2). In contrast, all paired cultures displayed lower activity than the corresponding monocultures in the DPPH assay. The strongest reduction was observed for the *Pleurotus* + *Pycnoporus* combination, whose scavenging capacity decreased by 44% and 68% compared with *P. ostreatus* and *P. sanguineus* grown alone.

The FRAP results followed a pattern similar to that observed in the other antioxidant assays, with lower iron-reducing capacity in most co-culture extracts (Table 2). The highest FRAP value was observed for the *T. polyzona* monoculture, exceeding the Trolox control by approximately 3.3-fold. High reducing capacity was also observed in the *P. sanguineus* monoculture and the *Pycnoporus* + *Trametes* co-culture, both of which exceeded the positive control. Despite this, the *Pycnoporus* + *Trametes* combination exhibited lower reducing activity than the respective monocultures, with reductions of 33% and 45% relative to *P. sanguineus* and *T. polyzona*, respectively.

In the β-carotene/linoleic acid assay, none of the extracts reached the inhibition level observed for the Trolox control (89.8%) (Table 2). Nevertheless, the *P. sanguineus* monoculture showed the highest inhibition of lipid peroxidation among the evaluated fungal systems. Co-cultivation generally reduced the protective effect against oxidative degradation when compared with the corresponding monocultures. The most significant reduction was observed for the *Lentinus* + *Pleurotus* combination, whose inhibitory capacity was reduced by 63% relative to each fungus cultivated alone.

Considering all the antioxidant assay results, they did not show the same response pattern across all extracts, suggesting that co-cultivation influenced the distinct antioxidant properties of the mycelial biomass.

### 2.3. Photoprotective Properties of Axenic and Co-Culture Extracts

The photoprotective responses of the axenic and paired culture extracts, including SPF and UVA/UVB ratio values, are presented in Table 3 and Table 4. The extracts showed concentration-dependent photoprotective activity, and several exhibited both high SPF values and strong UVA protection. The highest photoprotective responses were observed for the *P. ostreatus* and *T. polyzona* monocultures and for the *Pycnoporus* + *Trametes* co-culture.

*Pleurotus ostreatus* and *T. polyzona* showed high SPF (>39) maintained at the highest extract concentrations (Table 3). The paired culture *Lentinus* + *Pycnoporus* exhibited a higher SPF than the corresponding monocultures, exceeding 30 at 30 mg mL^−1^, and remained elevated at the highest concentrations. The *Pycnoporus* + *Trametes* co-culture showed higher SPF values than *P. sanguineus* monoculture. The other paired cultures showed distinct SPF responses depending on the fungal combination, with reductions more pronounced in the *Lentinus* + *Trametes* co-culture.

The UVA/UVB ratio increased with extract concentration in all evaluated systems (Table 4). Although differences among fungal combinations were observed, most extracts exhibited Ultra UVA protection at the highest concentrations. The highest UVA/UVB ratios were observed for the *P. ostreatus* monoculture, reaching 1.84 at 50 mg mL^−1^. High ratios were also found for the *Lentinus* + *Pycnoporus* and *Pycnoporus* + *Trametes* co-cultures, which remained classified as Ultra throughout most of the evaluated concentrations. Lower UVA/UVB values were observed for the *P. sanguineus* monoculture and for the *Lentinus* + *Pleurotus* co-culture, especially at lower concentrations, where the extracts were mainly classified as Good or Superior. At the same time, several paired cultures maintained strong UVA protection despite differences in SPF values.

The photoprotective characteristics of the extracts were not uniform among the fungal combinations. Some paired cultures maintained high UVA protection even when the SPF values differed from those observed for the fungi cultivated alone, suggesting that fungal interaction may influence distinct photoprotective properties of the extracts.

### 2.4. Anti-Tyrosinase Activity of Axenic and Co-Culture Extracts

**Figure 1:**
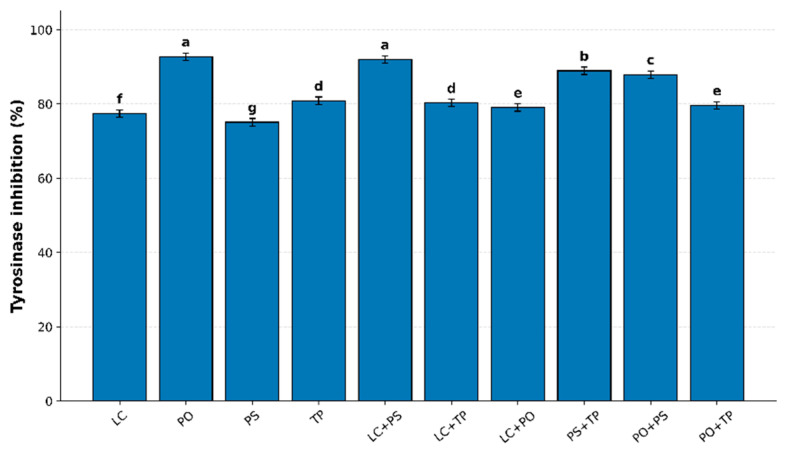
Tyrosinase inhibitory activity (%) of mycelial extracts obtained from axenic and paired cultures of basidiomycetes at 2 mg mL^−1^. Different lowercase letters indicate statistically significant differences among the extracts according to the Scott–Knott test (*p* ≤ 0.05). Values are expressed as mean ± standard deviation. LC = *Lentinus crinitus*, PO = *Pleurotus ostreatus*, PS = *Pycnoporus sanguineus*, and TP = *Trametes polyzona*.

All extracts showed high tyrosinase inhibitory activity, ranging from 75.0% to 92.7% (Figure 1). The highest inhibition percentages were observed in the *P. ostreatus* monoculture (92.7 ± 0.05%) and the *Lentinus* + *Pycnoporus* co-culture (92.0 ± 0.50%), both in the same statistical group. High inhibitory activity was also observed for the *Pycnoporus* + *Trametes* (88.9 ± 0.60%) and *Pleurotus* + *Pycnoporus* (87.9 ± 0.40%) co-cultures. Overall, some paired cultures exhibited stronger anti-tyrosinase activity than the corresponding monocultures, indicating that fungal interaction affected the inhibitory potential of the extracts. Differences in tyrosinase inhibition among the cultivations did not fully correspond to the antioxidant patterns observed in the previous assays, suggesting distinct responses associated with fungal interactions.

### 2.5. Molecular Target Prediction and GO Enrichment Analysis

To explore possible biological relationships associated with the compound profile identified in the extracts from the different mycelial cultures, molecular target prediction and enrichment analyses were performed.

Predicted molecular targets for the phenolic compounds identified in the fungal extracts varied between axenic and paired cultures. Flavonoids, particularly quercetin, catechin, and myricetin, were associated with a broader target profile than the remaining compounds. Subsequent enrichment analyses identified associations between these targets and biological processes related to oxidative stress, inflammation, apoptosis, cell signaling, and UV-associated cellular responses.

**Figure 2:**
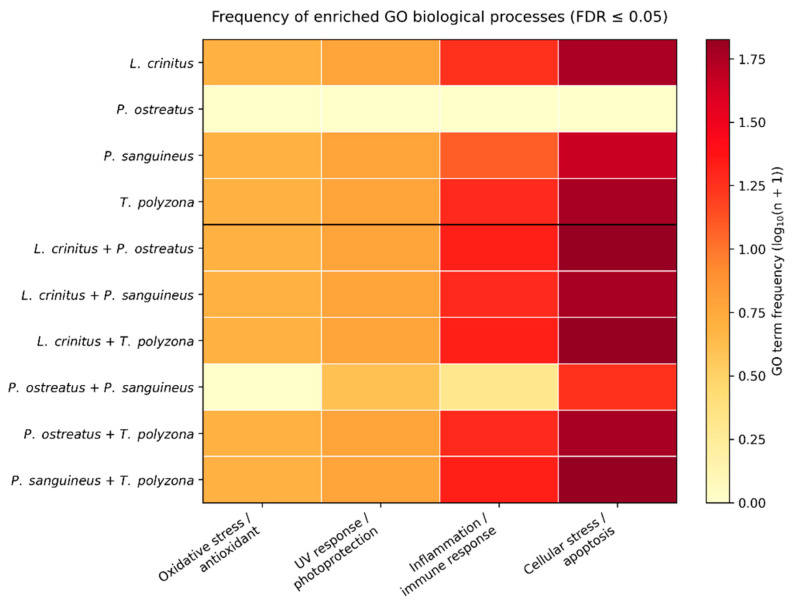
Heatmap showing the distribution of enriched Gene Ontology (GO) biological processes grouped into functional categories for axenic and paired cultures of the evaluated basidiomycetes. Values represent log10(n + 1)-transformed frequencies of significantly enriched GO terms (FDR ≤ 0.05).

Gene Ontology (GO) enrichment analysis showed differences among the axenic and paired cultures (Table 5 and Table 6; Figure 2). Many terms related to oxidative stress, UV response, inflammation, apoptosis, and cellular stress appeared repeatedly throughout the evaluated fungal cultivation. These findings showed partial correspondence with the antioxidant, photoprotective, and anti-tyrosinase in vitro assays.

Among the axenic cultures (Table 5), *L. crinitus*, *P. sanguineus*, and *T. polyzona* showed similar enrichment profiles, with significant GO terms related to response to oxidative stress, cellular response to oxidative stress, inflammatory response, regulation of inflammatory response, and regulation of apoptotic process. Terms associated with cellular response to UV-A were also identified for these extracts. In contrast, no statistically significant GO enrichment was observed for the *P. ostreatus* monoculture at the established significance threshold (FDR ≤ 0.05), despite its strong photoprotective and anti-tyrosinase activities observed in vitro.

The paired cultures also exhibited enriched GO terms associated with oxidative stress, UV response, inflammation, and apoptosis, although differences among fungal combinations were observed (Table 6). Co-cultures involving *L. crinitus* and *T. polyzona* concentrated a greater number of enriched biological processes and generally showed lower FDR values, especially for oxidative stress and UV-associated responses. In contrast, the *Pleurotus* + *Pycnoporus* combination showed fewer significantly enriched terms than the other paired cultures. The heatmap in Figure 2 summarizes the distribution of the functional categories identified in the evaluated systems, with cellular stress/apoptosis and inflammation/immune response appearing more frequently among the enriched terms.

The enrichment results observed among the fungal combinations were associated with differences in predicted cellular responses, mostly processes related to oxidative stress regulation and cellular stress adaptation.

### 2.6. KEGG Pathway Enrichment Analysis

**Figure 3:**
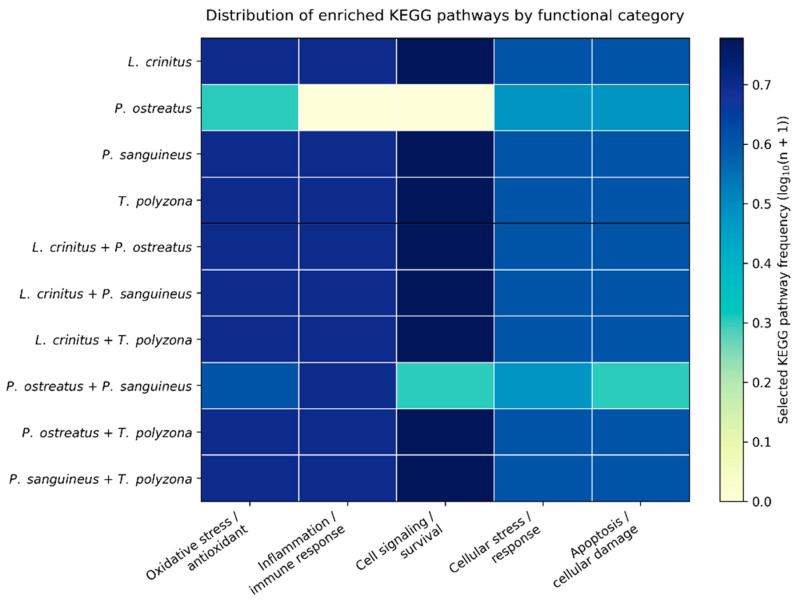
Heatmap showing the distribution of enriched KEGG pathways grouped into functional categories for axenic and paired cultures of the evaluated basidiomycetes. Values represent log10(n + 1)-transformed frequencies of enriched pathways.

KEGG (Kyoto Encyclopedia of Genes and Genomes) pathway enrichment analysis revealed pathways related to oxidative stress, inflammation, cell signaling, cellular stress response, and apoptosis in the fungal extracts obtained from axenic and paired cultures (Table S1 and Figure 3).

Among the axenic cultures, *L. crinitus*, *P. sanguineus*, and *T. polyzona* have several common pathways, including chemical carcinogenesis–reactive oxygen species, PI3K-Akt signaling, HIF-1 signaling, and FoxO signaling (Table S1). The p53 signaling pathway was detected in *P. sanguineus* and *T. polyzona*, whereas the *P. ostreatus* monoculture differed from the other fungi by the occurrence of HIF-1 signaling, autophagy, p53 signaling, and cysteine and methionine metabolism pathways.

Most paired cultures exhibited enrichment patterns similar to those observed for *L. crinitus*, *P. sanguineus*, and *T. polyzona*, with recurrent representation of PI3K-Akt, HIF-1, FoxO, p53, and oxidative stress-related pathways. An exception was the *Pleurotus* + *Pycnoporus* co-culture, which was associated with AGE-RAGE signaling, arachidonic acid metabolism, and lipid and atherosclerosis pathways, resulting in a greater representation of inflammation-related categories.

Figure 3 exhibits the distribution of the enriched KEGG pathways grouped into functional categories. Cell signaling/survival and oxidative stress categories were the most frequent, whereas inflammation-related pathways were more prominent in the *Pleurotus* + *Pycnoporus* co-culture.

### 2.7. Prediction of Biological Activities by PASS

Several antioxidant-related activities were predicted for the phenolic compounds identified in the basidiomycete extracts (Table 7). In addition to antioxidant activity and free radical scavenging, predictions were made for inhibition of lipid peroxidase, NADPH oxidase, and melanin.

Predicted antioxidant-related activities were observed for all identified compounds (Table 7). Catechin, quercetin, and myricetin stood out by presenting the highest Pa values for both antioxidant activity and free radical scavenging, whereas vanillin and the phenolic acids generally showed lower probabilities. Nevertheless, positive predictions were obtained for most compounds, suggesting that several of the identified phenolics may contribute to antioxidant-related activities.

Predictions related to lipid peroxidase inhibition were obtained for most of the identified compounds. The highest Pa values were observed for catechin, myricetin, quercetin, and vanillin, whereas phenolic acids showed moderate probabilities. Quercetin and myricetin showed the highest probabilities for NADPH oxidase inhibition, followed by gallic, syringic, and protocatechuic acids.

Predictions related to melanin inhibition were obtained for *p*-coumaric acid, catechin, quercetin, myricetin, and protocatechuic acid. The highest probabilities were observed for catechin, quercetin, and myricetin. Overall, the PASS results indicate that flavonoids had the broadest and highest predicted bioactivity spectrum among the identified phenolic constituents.

### 2.8. ADME Prediction

Quercetin and catechin met all drug-likeness criteria evaluated in this study (Table 8). Myricetin showed the largest number of violations, exceeding the recommended limits for TPSA and hydrogen-bond donor groups. In contrast, the phenolic acids and vanillin generally failed only the Ghose and Muegge filters, primarily due to their lower molecular weights. All compounds were predicted to be soluble or very soluble in water, and bioavailability scores remained within a relatively narrow range (0.55–0.85).

**Figure 4:**
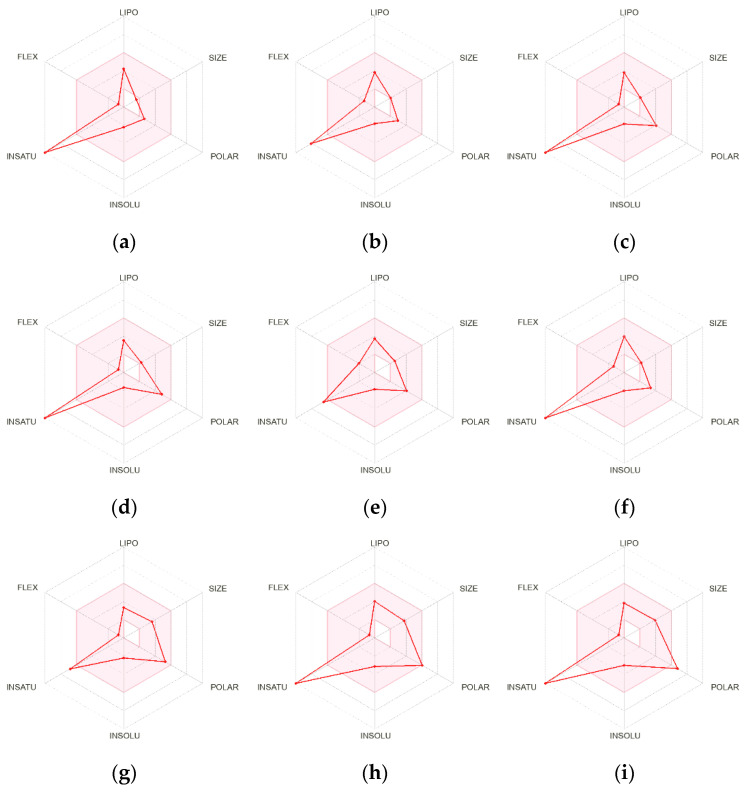
Bioavailability radar profiles generated by SwissADME for the phenolic compounds identified in basidiomycete mycelial extracts. The radar summarizes six physicochemical properties associated with oral bioavailability: lipophilicity, size, polarity, solubility, flexibility, and saturation. The pink area represents the ideal range for each property. (**a**) Benzoic acid; (**b**) vanillin; (**c**) protocatechuic acid; (**d**) gallic acid; (**e**) syringic acid; (**f**) *p*-coumaric acid; (**g**) catechin; (**h**) quercetin; and (**i**) myricetin.

The oral bioavailability radar highlighted differences among the identified phenolic compounds (Figure 4). None of the metabolites adjusted entirely within the optimal physicochemical space defined by the model. The most frequent deviation was related to the Csp^3^ fraction, which remained below the recommended range for all compounds. Myricetin also exceeded the recommended TPSA limit, whereas quercetin was positioned close to the upper boundary of the acceptable range.

**Figure 5:**
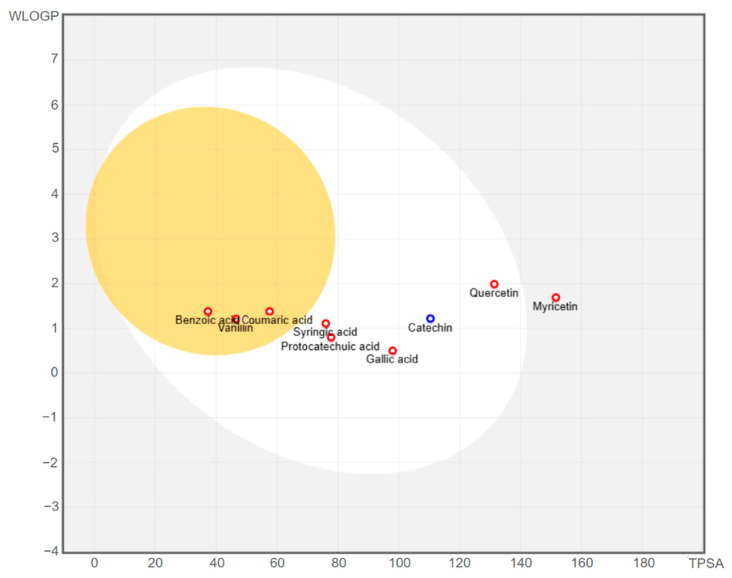
BOILED-Egg model generated by SwissADME for the phenolic compounds identified in basidiomycete mycelial extracts. Compounds located in the white region (egg white) are predicted to be passively absorbed by the gastrointestinal tract; those located in the yellow region (yolk) are predicted to permeate the blood–brain barrier (BBB). Blue dots indicate P-glycoprotein (P-gp) substrates and red dots indicate non-substrates.

The BOILED-Egg model distribution of the compounds is shown in Figure 5. Benzoic acid, vanillin, protocatechuic acid, gallic acid, syringic acid, *p*-coumaric acid, catechin, and quercetin were located within the region associated with high gastrointestinal absorption. Myricetin was the only compound positioned outside this area. Benzoic acid, vanillin, and *p*-coumaric acid were also predicted to cross the blood–brain barrier. Catechin was classified as a P-glycoprotein substrate, whereas the remaining compounds were predicted as non-substrates.

Predicted interactions with cytochrome P450 enzymes were observed for a subset of the identified compounds (Table 8). Quercetin was associated with CYP1A2, CYP2D6, and CYP3A4 inhibition. Myricetin showed predicted inhibition of CYP1A2 and CYP3A4, whereas protocatechuic and gallic acids were associated only with CYP3A4. No CYP inhibition was predicted for benzoic acid, vanillin, syringic acid, *p*-coumaric acid, or catechin.

Benzoic acid was associated with the highest predicted skin permeability (Log Kp = −5.72 cm/s), whereas catechin showed the lowest value (Log Kp = −7.87 cm/s). The other compounds were distributed between these limits (Table 9).

### 2.9. Toxicity Prediction

The toxicity profiles predicted by ProTox-III are presented in Table 9. Predicted oral LD_50_ values ranged from 159 to 10,000 mg/kg body weight. Catechin showed the highest LD_50_ value and was classified as toxicity class VI (non-toxic), whereas *p*-coumaric acid was assigned to class V (may be harmful if swallowed). Vanillin, protocatechuic acid, gallic acid, and syringic acid were classified as class IV (harmful if swallowed). Benzoic acid, quercetin, and myricetin presented the lowest predicted LD_50_ values and were classified as toxicity class III (toxic if swallowed).

Nephrotoxicity was predicted for all evaluated compounds, with probabilities ranging from 53% to 69% (Table 9). Respiratory toxicity alerts were observed for gallic acid, catechin, quercetin, and myricetin, whereas hepatotoxicity was predicted only for benzoic acid. Most compounds were predicted as inactive for neurotoxicity, cardiotoxicity, immunotoxicity, and cytotoxicity. Carcinogenicity alerts were predicted for protocatechuic acid, gallic acid, quercetin, and myricetin, while mutagenicity was predicted only for quercetin and myricetin.

## 3. Discussion

Co-cultivation altered the phenolic composition of the mycelial extracts in a manner that depended on the fungal combination. Although most paired cultures exhibited lower total phenolic content than their corresponding monocultures, several interactions promoted the appearance of compounds not detected under axenic conditions. Catechin and syringic acid were identified exclusively in selected co-cultures, whereas *p*-coumaric acid emerged during the interaction between *P. ostreatus* and *P. sanguineus*. These findings indicate that fungal interactions influenced the qualitative composition of phenolics more strongly than total phenolic accumulation. Previous studies have shown that interactions between fungi may modify secondary metabolism through competitive or signaling processes, leading to changes in metabolite profiles [31]. In the present study, the emergence of phenolics absent in axenic cultures suggests that co-cultivation altered metabolic regulation, leading to the induction or suppression of specific compounds. Some of the compounds induced during co-cultivation, including catechin and *p*-coumaric acid, have been associated with antioxidant, photoprotective, and tyrosinase-inhibitory activities. Therefore, changes in phenolic composition may have biological consequences that extend beyond simple differences in total phenolic content. Although the underlying mechanisms were not investigated, the observed shifts in phenolic composition indicate that interspecific interactions can substantially reshape the chemical profile of fungal mycelia.

Gallic acid was the only phenolic compound detected in all evaluated extracts, indicating that its production was maintained regardless of the fungal species or cultivation system. In contrast, quercetin occurrence varied among the cultures and was absent from the *P. ostreatus* monoculture and the *Pleurotus* + *Pycnoporus* co-culture. The exclusive detection of catechin and syringic acid in paired cultures is particularly noteworthy, as these compounds were not produced when the fungi were cultivated separately. The paired culture *Pleurotus* + *Pycnoporus* induced *p*-coumaric acid production, whereas the highest concentration of this compound was observed in *Lentinus* + *Pycnoporus*. The co-cultivation of *Lentinus* + *Pleurotus* increased the concentration of protocatechuic acid compared with the respective monocultures. In general, the remaining co-culture combinations reduced, inhibited, or had no effect on phenolic compound concentrations. Thus, the effects of co-cultivation on phenolic production depended on the fungal species involved. The increase or decrease in individual compounds may reflect biochemical responses triggered during mycelial interactions.

Sułkowska-Ziaja et al. [33] evaluated the concentrations of 5 phenolic compounds in 6 *Ganoderma* species and found marked differences among species. *Ganoderma applanatum* presented all the evaluated compounds, while *G. resinaceum* and *G. carnosum* contained only three. In contrast to the present study, protocatechuic acid was the only compound detected in all species. Carvajal et al. [34] identified pyrogallol, gallic, protocatechuic, and benzoic acids in both the mycelium and fruiting body of *Agaricus brasiliensis*, although their concentrations varied according to the developmental stage. Together, these studies show that phenolic composition varies among fungal species and developmental stages. Comparisons with the present study should be made with caution, as previous investigations have primarily evaluated monocultures rather than fungal co-cultivation systems.

The diversity of phenolics in the mycelium extracts varied. While *L. crinitus* presented the largest number of identified phenolic compounds, it showed the lowest total phenolic content among the axenic cultures. The opposite was observed for *P. sanguineus*, which presented the highest total phenolic content and a less diverse phenolic profile. This contrast was also evident in some paired cultures. Nowacka et al. [35] reported similar findings, in which *Fomes fomentarius* exhibited the highest total phenolic content among the evaluated mushrooms, despite *Hyphodontia paradoxa* containing a broader range of phenolic acids. These observations show that extracts rich in total phenolics are not necessarily those with the greatest diversity of identified compounds. It should also be considered that the present study focused exclusively on phenolic compounds associated with the mycelial biomass. Metabolites released into the culture medium were not evaluated and may also contribute to the biological properties of fungal cultures.

Changes in the phenolic profile of the extracts may also influence their biological properties. For this reason, the antioxidant activity of the monocultures and paired cultures was evaluated in relation to both the total phenolic content and the individual compounds identified by chromatographic analysis.

The highest antioxidant activity among the evaluated fungi was detected for *P. sanguineus* and *T. polyzona* monocultures, regardless of the assay employed. These species also exhibited high concentrations of phenolic compounds, with *P. sanguineus* having the highest total phenolic content. Krupodorova et al. [36] reported a positive correlation between phenolic content and antioxidant activity for mycelial extracts of 30 macrofungi cultivated under submerged conditions. Nevertheless, the antioxidant activity of the extracts cannot be interpreted solely on the basis of total phenols, since other compounds with antioxidant activity, such as terpenoids, organic acids, and polysaccharides, are produced by fungi [37]. Krupodorova et al. [36] also observed associations between antioxidant activity and both phenolic compounds and polysaccharides in mycelial extracts.

The antioxidant response of the paired cultures differed from the changes observed in phenolic composition. Although co-cultivation altered the phenolic profile and led to the emergence of compounds not detected in monocultures, most paired cultures exhibited lower antioxidant activity than their corresponding axenic cultures. The decrease in antioxidant activity was consistently observed across all assays, despite the different reaction mechanisms involved. This pattern indicates that co-cultivation affected multiple components that contribute to the antioxidant capacity of the extracts, rather than a specific antioxidant pathway. Therefore, the changes in phenolic composition observed in paired cultures were not necessarily associated with improved antioxidant performance, highlighting the importance of the balance and relative abundance of bioactive metabolites rather than the presence of individual compounds alone. Similar discrepancies between phenolic content and antioxidant activity have been reported in other fungal interaction systems. When Chen et al. [38] co-cultivated *Cordyceps takaomontana* with two soil fungi, for example, an increase in antioxidant activity in co-cultures was observed despite a reduction in total phenolic content in one interaction. The same cultures showed increased triterpenoid accumulation, suggesting that fungal interactions may simultaneously affect different groups of antioxidant metabolites.

Gallic acid and quercetin were among the most abundant phenolic compounds detected in the extracts. The compounds are associated with antioxidant activity in mushrooms and other natural products [39,40]. Catechin and syringic acid, detected only in some paired cultures, are also known for their radical-scavenging and reducing-agent properties [41]. However, the presence of these compounds alone was insufficient to explain the antioxidant responses observed among the extracts, since co-cultivation frequently reduced antioxidant activity despite changes in phenolic composition. Nevertheless, the induction of catechin in selected co-cultures indicates that fungal interactions may favor the synthesis of compounds with antioxidant properties, even when the resulting extracts do not exhibit higher overall antioxidant activity.

In addition to their antioxidant properties, phenolic compounds have been associated with absorption of ultraviolet radiation and protection against photoinduced oxidative damage [42]. Therefore, the photoprotective potential of the axenic and paired-culture extracts was also evaluated.

Mycelial extracts exhibited pronounced photoprotective activity, particularly at the highest concentrations. *P. ostreatus* and *T. polyzona* monocultures showed the highest SPF values (~39), while the *Lentinus* + *Pycnoporus* and *Pycnoporus* + *Trametes* co-cultures also displayed high photoprotective performance. In addition, most extracts were classified as Ultra at the highest concentrations according to the Boots star rating system. These findings highlight the potential of basidiomycete mycelium as a source of compounds with broad-spectrum photoprotective properties.

The high SPF values observed for the extracts are consistent with previous reports. Wontcheu Fotso et al. [14] reported SPF values above 30 for extracts of *Inonotus obliquus* rich in polyphenols and flavonoids. In contrast, lower SPF values have been reported for mycelial extracts of other basidiomycetes evaluated by the same spectrophotometric method, including *Trametes versicolor* (SPF 2.17), *Laetiporus sulphureus* (SPF 3.30), and *Ganoderma applanatum* (SPF 9.03) [43]. In the present study, gallic acid, quercetin, catechin, and *p*-coumaric acid were detected among the phenolic constituents of the extracts. Among these compounds, catechin and *p*-coumaric acid have been associated with UV absorption and protection against photoinduced oxidative damage, which may have contributed to the photoprotective responses observed in some co-cultures [44]. The high SPF values observed for some extracts indicate substantial UV absorption; however, SPF determination under spectrophotometric conditions provides information on the optical properties of the extracts and does not account for factors that influence photoprotective performance in topical formulations.

Unlike its effect on antioxidant activity, co-cultivation did not negatively affect the photoprotective activity of the extracts. Some paired cultures, such as *Lentinus* + *Pycnoporus* and *Pycnoporus* + *Trametes*, showed SPF values as high as their respective monocultures. This suggests that interactions between species during co-cultivation affect antioxidant and photoprotective activities in distinct ways.

In addition to UVB protection, the extracts also presented UVA protection, a desirable characteristic in photoprotection. The paired cultivation of *Lentinus* + *Pycnoporus* expanded the photoprotective spectrum. Even at the lowest concentration tested, where the co-culture extract already statistically exceeded its respective monocultures’ SPF, the UVA/UVB ratio reached the “ultra” protection level (Table 3 and Table 4) according to Boots’ criteria [32]. This may indicate that the biotic stress generated by the mycelial confrontation [5] not only stimulated the biosynthetic pathway of chromophores that confer UVB protection, but also induced the accumulation of metabolites such as quercetin, whose production was significantly increased in *Lentinus* + *Pycnoporus* (Table 1), capable of absorbing longer wavelengths [42].

On the other hand, some co-cultures reduced the amplitude of UVA protection, as in the *Lentinus* + *Trametes* and *Lentinus* + *Pleurotus* combinations. Both also showed a reduction in antioxidant activity and in the diversity of phenolic compounds. This set of modifications indicates that metabolic alterations depend on the species in the co-culture, and the result may be greater or lesser photoprotective activity.

The mycelial biomass of the evaluated basidiomycetes contains compounds capable of acting directly in cutaneous photoprotection. The fact that these SPF and UVA absorption indices were achieved using crude extracts, without the need for complex molecular purification steps, highlights the potential of fungal cultivation as a source of bioactive compounds for cosmetic applications [16,45]. However, the practical use of these extracts in photoprotective formulations requires further investigation, including studies on photostability, safety, formulation compatibility, and biological efficacy. Therefore, co-cultivation may represent a useful biotechnological strategy for generating metabolically diverse extracts with potential for future cosmetic development.

Regarding tyrosinase inhibition, all extracts showed high tyrosinase inhibitory activity, with particular emphasis on the monoculture of *P. ostreatus* and the paired culture *Lentinus* + *Pycnoporus*, which promoted inhibition close to 100%. However, the results suggest that the tyrosinase inhibition observed in the fungal extracts occurs through distinct phytochemical pathways that depend on the cultivation system. In the *P. ostreatus* monoculture, the inhibition appears to be mediated by its high benzoic acid content, a class whose p-hydroxybenzoic derivative acts specifically as a reversible competitive inhibitor at the enzyme’s active site [46]. In the *Lentinus* + *Pycnoporus* co-culture, the same level of enzyme inhibition appears to have occurred through an alternative synergistic mechanism. Although the benzoic acid content was reduced, there was a significant increase in the *p*-coumaric acid content. *p*-Coumaric acid also acts as a potent competitive inhibitor of tyrosinase due to its structural analogy with the natural substrate L-tyrosine [15]. This induction, associated with the significant accumulation of quercetin in the pair, demonstrates that co-culture stress can activate distinct metabolic pathways. These observations are reinforced when the anti-tyrosinase activity of the *P. sanguineus* monoculture is evaluated. In this extract, no benzoic or *p*-coumaric acid was detected, resulting in the lowest anti-tyrosinase activity. However, the inhibitory effect might be due to the content of protocatechuic acid and quercetin [47]. In addition, catechin, detected exclusively in selected co-cultures, has been associated with tyrosinase inhibition and modulation of melanogenesis-related pathways [48], suggesting that multiple phenolics may have contributed to the inhibitory activity observed in paired cultures.

The photoprotective and anti-tyrosinase activities observed in the present study are interesting when considered together. Exposure to UV radiation is one of the main factors stimulating melanogenesis, a process in which tyrosinase plays a central role [44]. In this context, a combination of UV protection and tyrosinase inhibition may help control photoinduced skin pigmentation. Similar approaches have been explored for fungal-derived ingredients for cosmetic applications, including studies with mycelial extracts exhibiting both photoprotective and anti-tyrosinase properties [43,49]. The combination of these activities in basidiomycete extracts increases their potential for dermocosmetic applications.

The in vitro assays showed that the fungal extracts combined antioxidant activity, photoprotective potential, and tyrosinase inhibition. Since these effects may involve related cellular responses, GO biological processes and KEGG pathways associated with the experimental endpoints were examined among the predicted molecular targets. The selected categories included processes related to oxidative stress, UV response, inflammation, apoptosis, and cellular adaptation to stress. Thus, the computational analyses were used as supporting evidence to interpret the biological activities observed experimentally.

Among the selected GO categories, processes related to oxidative stress and UV response were frequent in both axenic and paired cultures. Terms such as response to oxidative stress, cellular response to oxidative stress, and cellular response to UV-A were consistent with the experimental profiles of the extracts, particularly those exhibiting high antioxidant activity, elevated SPF values, and broad-spectrum UV protection. This association is biologically plausible because UV radiation can affect cellular redox homeostasis and activate stress-response pathways, whereas phenolic compounds may modulate these processes by multiple cellular targets [50,51].

GO categories related to inflammation, apoptosis, and cellular stress also appeared among the selected terms. These processes occurred together with oxidative stress- and UV-associated categories, which are relevant because UV-induced damage often involves redox imbalance, inflammatory signaling, and apoptotic responses [51]. Although inflammation and apoptosis were not evaluated experimentally, their presence among the selected GO terms suggests that some identified phenolics may be linked to broader cellular responses to environmental stress.

The KEGG analysis identified pathways associated with the same biological themes highlighted by the GO categories, including oxidative stress responses, apoptosis, cell survival, and cellular adaptation to stress. These pathways were present in most fungal cultures and were consistent with the antioxidant and photoprotective activities observed experimentally.

Among the recurrent KEGG pathways, PI3K-Akt (Phosphatidylinositol 3-kinases/protein kinase B) and FoxO (forkhead box subfamily of transcription factors) signaling were represented in both axenic and paired cultures. These pathways are involved in cellular responses to UV-induced damage, oxidative stress, and redox regulation [51,52]. Their occurrence across multiple fungal systems was consistent with experimentally observed antioxidant and photoprotective activities and suggests that some of the identified phenolic compounds may interact with molecular targets associated with cellular stress responses.

HIF-1 (Hypoxia-Inducible Factor 1) and p53 (tumor suppressor protein) signaling pathways were also identified in both axenic and paired cultures. The p53 pathway participates in cellular responses to UV-induced damage and apoptosis [53,54], whereas HIF-1 signaling is associated with adaptation to oxidative and inflammatory stress [55]. The occurrence of these pathways, together with GO categories related to oxidative stress, UV response, inflammation, and apoptosis, indicates that similar biological functions were represented in both enrichment analyses. Although these associations were derived from computational predictions, they were compatible with the antioxidant, photoprotective, and tyrosinase-inhibitory activities observed experimentally.

PASS predictions were generally compatible with experimentally observed biological activities and highlighted differences among the identified phenolic compounds [56,57]. Antioxidant activity, free radical scavenging, inhibition of lipid peroxidation, inhibition of NADPH oxidase, and melanin inhibition were among the most frequently predicted activities.

All identified phenolic compounds exhibited predicted antioxidant-related activities, with the highest probabilities observed for catechin, quercetin, and myricetin. Catechin deserves particular attention because it was detected exclusively in paired cultures and showed high probabilities for antioxidant activity, free radical scavenging, lipid peroxidase inhibition, and melanin inhibition. Flavonoids were generally associated with the broadest spectrum of predicted activities [58], whereas vanillin and most phenolic acids showed lower probabilities. These predictions were compatible with the experimentally observed antioxidant and tyrosinase-inhibitory activities and suggest that flavonoids may contribute to the biological properties of the fungal extracts.

Most identified phenolic compounds were predicted to have good aqueous solubility and high gastrointestinal absorption. Catechin and quercetin fulfilled all drug-likeness criteria, whereas myricetin showed less favorable properties because of its higher polarity [59]. Predicted interactions with P-glycoprotein and cytochrome P450 enzymes were limited for most metabolites. These predictions provide complementary information on the physicochemical and pharmacokinetic characteristics of the identified phenolic compounds [60].

The toxicity predictions varied among the identified phenolics. Catechin showed the highest predicted LD_50_ and was the only compound classified as non-toxic, whereas quercetin, myricetin, and benzoic acid were assigned to toxicity class III. Moderate probabilities of nephrotoxicity were predicted for all metabolites, while respiratory toxicity and carcinogenicity were limited to specific compounds. These results suggest that the safety profile may differ substantially among the phenolics detected in the fungal extracts [61]. As the compounds were evaluated individually, interactions among metabolites present in the extracts were not considered. Therefore, the predictions describe the toxicological potential of the identified phenolics rather than the safety of the extracts as a whole.

In summary, co-cultivation modified the phenolic composition of the fungal extracts and led to the emergence of compounds not detected in axenic cultures. These changes were accompanied by antioxidant, photoprotective, and tyrosinase-inhibitory activities, highlighting the potential of fungal interactions as a strategy for obtaining bioactive metabolites of cosmetic interest. The computational analyses provided complementary information that helped contextualize the experimentally observed biological activities and identify potential biological processes and pathways associated with the detected phenolic compounds. Further studies will contribute to the validation and application of these fungal metabolites.

## 4. Materials and Methods

### 4.1. Fungal Strains and Inoculum Production

Four basidiomycete strains, *Lentinus crinitus* U9-1, *Pleurotus ostreatus* U12-4, *Pycnoporus sanguineus* U13-4, and *Trametes polyzona* U16-5 from the Paranaense University culture collection were used in this study. The mycelial biomass was grown in 20 g L^−1^ malt extract-agar (MEA) at 28 ± 1 °C for 7 days. Mycelial plugs (6 mm diameter) obtained from actively growing MEA cultures were used as inoculum for axenic and co-culture experiments.

### 4.2. Mycelial Biomass Production in Axenic and Co-Culture Systems

Axenic and paired cultures were established as described by Avelino et al. [62]. The fungi were cultivated in 250 mL Erlenmeyer flasks containing 100 mL of malt extract medium (20 g L^−1^), previously sterilized at 121 °C for 20 min. The inoculum consisted of six MEA discs with mycelium, three of each fungus in the co-cultures, and six of one species in monocultures. All the flasks were incubated at 28 ± 1 °C without agitation for 22 days. The cultivation period was selected based on previous studies using the same fungal strains under similar conditions [62]. The mycelial biomass was recovered by filtration (Whatman grade 1 filter paper) and dried at 60 °C until constant weight for biomass determination. After drying, the entire mycelial biomass from each culture was macerated in a mortar under liquid nitrogen until a fine, homogeneous powder was obtained. The resulting powder was then used for the extraction procedures. Dry biomass production data are presented in Supplementary Table S2.

### 4.3. Preparation of Mycelial Biomass Extracts

Bioactive compounds from the mycelial biomass (3 g) were extracted with 15 mL of 80% ethanol for 45 min at 50 °C [63]. Next, the mixture was centrifuged at 4400× *g* at 10 °C for 20 min, and the supernatant was recovered and re-extracted as described previously. Finally, the supernatants were combined and stored at −80 °C for further analysis.

### 4.4. Total Phenolic Content

The total phenolic content in the mycelial biomass extracts was determined as described by Singleton et al. [64]. The extracts (100 mg mL^−1^) were diluted in 80% ethanol up to 40, 60, and 80 mg mL^−1^. Samples (0.3 mL) of the extracts were mixed with 2.5 mL of 10% Folin–Ciocalteu reagent and 2.0 mL of 7.5 g L^−1^ sodium carbonate. The mixture was incubated for 15 min at 50 °C, and the absorbance was determined at 760 nm (SpectraMax Plus 384, Molecular Devices, San Jose, USA). A mixture of ethanol and water (4:1) was used as an analytical control. Total phenolic content was calculated against a gallic acid standard curve (10–700 µM). The results were expressed as µg of gallic acid equivalents (GAE) per mg of mycelial biomass (dry basis).

### 4.5. Phenolic Compounds Determination

Phenolic compounds were assessed using an HPLC system (LC-20A Prominence, Shimadzu, Kyoto, Japan) coupled with a UV-Vis detector (SPD-20A, Shimadzu, Kyoto, Japan), according to Palacios et al. [65]. Separation was conducted on a reverse-phase C-18 column (25 cm × 4,6 mm × 5 µm, Shim-pack CLC-ODS (M), Shimadzu, Kyoto, Japan). The solvents used were 0.1% acetic acid in water (solvent A) and 0.1% acetic acid in acetonitrile (solvent B) [62]. The elution (flow rate 1 mL min^−1^) gradient consisted of 100% A; 95% A and 5% B over 2 min; 70% A and 30% B over 25 min; 10% A and 90% B over 23 min; 100% B over 10 min; and 100% A over 3 min. The column temperature was maintained at 40 °C. The extracts (40 mg mL^−1^) were previously filtered (0.22 µm pore membrane), and a 20 μL sample was injected in duplicate into the equipment. The phenolic compounds in hydroalcoholic extracts were detected at 280 and 320 nm, characterized by their UV spectra and retention times, and compared with authentic standards. Quantification was performed using external calibration curves prepared from authentic standards of gallic acid, protocatechuic acid, syringic acid, *p*-coumaric acid, benzoic acid, catechin, quercetin, myricetin, and vanillin as described by Fernandes et al. [66]. HPLC quantification was performed using two independent biological replicates for each treatment, and each extract was analyzed in duplicate injections. The results were expressed as μg per g (dry basis). Calibration curves, coefficients of determination (R^2^), limits of detection (LOD), and limits of quantification (LOQ) for all standards are presented in Supplementary Table S3. Representative chromatograms of standards and extracts are shown in Supplementary Figures S1–S11.

### 4.6. Antioxidant Activity of Mycelial Biomass Extracts

The mycelial biomass extracts were diluted in 80% ethanol up to 40, 60, 80, and 100 mg mL^−1^ and used in the determination of the antioxidant activity by DPPH (2,2-diphenyl-1-picrylhydrazyl) free radical scavenging assay, ferric reducing antioxidant power (FRAP) assay, and β-carotene/linoleic acid co-oxidation (BCLA) method.

#### 4.6.1. DPPH Radical-Scavenging Assay

An aliquot (0.01 mL) of each extract dilution was mixed with 0.29 mL of 60 μM DPPH prepared in methanol on a 96-well plate. The mixture was kept for 30 min at 22 °C in the dark. The change in absorbance was determined at 515 nm (SpectraMax Plus 384, Molecular Devices, San Jose, CA, USA). An analytical control was prepared by replacing the extract with ethanol. The concentration required to reduce 50% of DPPH free radicals (IC_50_) was determined from concentration–response curves [10]. Quercetin, used as a positive control, was evaluated at concentrations ranging from 0 to 60 μM (in 10 μM increments), and its IC_50_ value was calculated using the same approach.

#### 4.6.2. Ferric Reducing Antioxidant Power (FRAP) Assay

Initially, the FRAP reagent, containing 25 mL of acetate buffer (300 mM, pH 3.6), 2.5 mL of 10 mM TPTZ (2,4,6-tris(2-pyridyl)-s-triazine) aqueous solution, and 2.5 mL of 20 mM ferrous chloride aqueous solution, was prepared. A sample (0.01 mL) of the extract dilutions was mixed with 0.29 mL of FRAP reagent on a 96-well plate. The reaction was incubated at 35 °C for 30 min, and the absorbance was measured at 595 nm using the same equipment described above. The percentage of antioxidant activity was calculated by comparing it with a standard curve of ferrous sulfate (500–2000 μM). Trolox was used as a positive control [10].

#### 4.6.3. β-Carotene/Linoleic Acid (BCLA) Assay

Aliquots (0.02 mL) of each extract dilution were mixed with 0.280 mL of a β-carotene-linoleic acid mixture in a 96-well plate. The reaction was incubated at 35 °C. The absorbance was determined at 470 nm using the same equipment mentioned earlier, every 10 min for up to 240 min of the reaction. Trolox was used as a positive control. The results were expressed as a percentage of inhibition of β-carotene oxidation. The reduction in absorbance of the system in the absence of antioxidants was considered 100% oxidation [10].

### 4.7. Photoprotective Activity

#### 4.7.1. Determination of Sun Protection Factor (SPF)

The extract was used to determine the sun protection factor, as described by Oliveira et al. [67]. The extract was diluted in ethanol: water (80:20, *v*/*v*) at concentrations of 10, 20, 30, 40, and 50 mg mL^−1^. The absorbance of each dilution was determined in triplicate at wavelengths from 290 to 320 nm in 5 nm increments using a SpectraMax Plus 384 microplate reader (Molecular Devices, San Jose, CA, USA). An ethanol: water (80:20, *v*/*v*) solution was used as the blank.

SPF values were calculated according to Equation (1):

(1) $$SPF=CF\sum_{290}^{320}EE\left(\lambda \right)I\left(\lambda \right)Abs(\lambda )$$

where CF is the correction factor (10), EE(λ) is the erythemal effect spectrum, I(λ) is the solar intensity spectrum, and Abs(λ) is the absorbance of the sample at wavelength λ [68].

#### 4.7.2. Determination of the UVA/UVB Ratio

The UVA/UVB ratio was calculated from the mean absorbance values in the UVA (320–400 nm) and UVB (290–320 nm) regions, as described by Wu et al. [69]. Higher UVA/UVB ratios indicate greater UVA protection relative to UVB protection. The absorption spectrum of each dilution of the extract was determined in triplicate at wavelengths from 290 to 400 nm, with increments of 5 nm, using a SpectraMax Plus 384 microplate reader (Molecular Devices, San Jose, CA, USA). Ethanol was used as an analytical control. The UVA/UVB ratios were calculated according to Equation (2):

(2) $$UVA/UVB=\frac{\int_{320}^{400}A\left(\lambda \right)d\lambda /\int_{320}^{400}d\lambda }{\int_{290}^{320}A\left(\lambda \right)d\lambda /\int_{290}^{320}d\lambda }$$

where A(λ) is the average absorbance at each wavelength, and dλ is the wavelength interval between measurements. The extracts were classified according to the Boots Star Rating System [43] as follows: <0.20, too low for a UVA claim; 0.21–0.40, minimum; 0.41–0.60, moderate; 0.61–0.80, good; 0.81–0.90, superior; and >0.90, ultra.

### 4.8. Tyrosinase Inhibitory Activity

Tyrosinase inhibitory activity was evaluated by a microscale colorimetric assay using L-DOPA (L-3,4-dihydroxyphenylalanine) as substrate. For the assay, the mycelial extracts were dissolved in 50% (*v*/*v*) DMSO at concentrations of 10, 20, 30, 40, and 50 mg mL^−1^, corresponding to final concentrations of 2, 4, 6, 8, and 10 mg mL^−1^ in the reaction mixture. Preliminary assays showed high tyrosinase inhibition across the tested concentration range (2–10 mg mL^−1^), with inhibition values consistently exceeding 70%. Therefore, 2 mg mL^−1^ was selected as the lowest effective concentration for comparative analyses among treatments. The reaction system was established in 96-well microplates using 100 mM phosphate buffer (pH 6.8). Each well received 80 µL of phosphate buffer, 40 µL of extract solution, and 40 µL of tyrosinase solution (250 U mL^−1^). The mixture was pre-incubated at 30 °C for 15 min prior to the addition of the substrate L-DOPA (5 mM) [70]. A second incubation was carried out under the same conditions for the chromogenic development of dopachrome. Absorbance readings were performed at 475 nm using a microplate reader (SpectraMax Plus 384, Molecular Devices, San Jose, CA, USA). Reaction mixtures without extract (enzyme control) and without substrate (blank) were used as analytical controls. Analyses were conducted in triplicate, and the percentage of inhibition was calculated according to Equation (3):

(3) $$\mathrm{Inhibition}\;(\%)=\left(\frac{AbsC-AbsE}{AbsC}\right)\times 100$$

where AbsC is the absorbance of the control reaction, and AbsE is the absorbance of the reaction containing the extract.

### 4.9. Molecular Target Prediction, Gene Ontology, and KEGG Enrichment Analyses

Phenolic compounds identified by HPLC in the mycelial extracts were used to predict molecular targets. Canonical SMILES (Simplified Molecular Input Line Entry System) were obtained from PubChem https://pubchem.ncbi.nlm.nih.gov/ (accessed on 20 April 2026) and submitted to the SwissTargetPrediction platform [71,72] to identify *Homo sapiens* putative molecular targets associated with each chemical profile. The predicted targets obtained for each axenic and co-culture extract were compiled and used for functional enrichment analysis.

To identify biological processes potentially associated with antioxidant activity, oxidative stress response, inflammation, UV response, apoptosis, and cellular stress-related mechanisms, Gene Ontology (GO) biological processes enrichment was performed using PANTHER [73]. Additionally, to identify metabolic and signaling pathways associated with the compounds detected in the extracts, KEGG pathway enrichment analysis was performed using Enrichr [74]. Only significantly enriched GO terms and KEGG pathways were considered for interpretation (false discovery rate, FDR ≤ 0.05 or adjusted *p*-value ≤ 0.05).

Biologically relevant and representative terms were selected, whereas broader or poorly informative terms were not considered in order to reduce redundancy in the analysis. The resulting GO terms and KEGG pathways were then organized according to their main biological associations, including oxidative stress and antioxidant response, inflammation and immune response, apoptosis and cell damage, cellular stress, signaling pathways, and cell survival. Heatmaps were generated from the frequencies of selected GO terms and KEGG pathways within each functional category, using a log10(n + 1) transformation.

### 4.10. Prediction of Biological Activities (PASS)

The biological activities of the phenolic compounds identified in the mycelial extracts were predicted using the PASS Online platform (Prediction of Activity Spectra for Substances; https://www.way2drug.com/passonline/ (accessed on 20 April 2026). Canonical SMILES (Simplified Molecular Input Line Entry System) of each identified compound in the different basidiomycete extracts were obtained from PubChem and submitted individually to PASS Online [57].

The software predicts the biological activity of natural or synthetic compounds based on structure–activity relationships by considering pharmacological characteristics, mechanisms of action, metabolic interactions, and enzyme-associated transport processes [56]. PASS estimates the probabilities of activity (Pa) and inactivity (Pi) for each predicted biological activity by comparing the submitted chemical structures with a database of known bioactive compounds. A prediction was considered active when the Pa value exceeded the corresponding Pi value [56]. Pa and Pi values range from 0.000 to 1.000.

Based on the biological properties experimentally evaluated in this study, predicted activities associated with antioxidant potential and regulation of melanogenesis were selected for further analysis, including antioxidant activity, free radical scavenging, lipid peroxidase inhibition, NADPH oxidase inhibition, and melanin inhibition. Only activities with Pa > Pi were considered biologically relevant. Predicted activities were interpreted according to the PASS recommendations: Pa > 0.70 indicates a high probability of experimental activity; 0.50 < Pa ≤ 0.70 indicates a moderate probability; and Pa ≤ 0.50 indicates a lower probability of activity.

### 4.11. In Silico Pharmacokinetic Analysis (ADME)

The pharmacokinetic properties of the phenolic compounds identified in the mycelial extracts were predicted using the SwissADME online tool http://www.swissadme.ch/ (accessed on 21 April 2026) [59]. The ADME (absorption, distribution, metabolism, and excretion) properties of the compounds were predicted to provide information on pharmacokinetics, drug similarity, and medicinal chemistry [59]. Canonical SMILES of the compounds were submitted individually to evaluate physicochemical properties, pharmacokinetic behavior, and drug-likeness.

The analysis included molecular descriptors such as molecular weight, fraction Csp^3^, number of rotatable bonds, hydrogen-bond donors and acceptors, molar refractivity, topological polar surface area (TPSA), lipophilicity (consensus LogP), and aqueous solubility (LogS). Pharmacokinetic parameters related to gastrointestinal absorption, blood–brain barrier permeation, P-glycoprotein substrate prediction, inhibition of cytochrome P450 isoforms (CYP1A2, CYP2C9, CYP2C19, CYP2D6, and CYP3A4), skin permeability (Log Kp), and bioavailability score were also obtained.

Drug-likeness and oral bioavailability were evaluated using the criteria implemented in SwissADME, including the Lipinski, Ghose, Veber, Egan, and Muegge filters. These filters assess the suitability of compounds for oral administration based on physicochemical properties such as molecular weight, lipophilicity, polarity, hydrogen-bonding capacity, molecular refractivity, and molecular flexibility. The Bioavailability Radar and BOILED-Egg models were additionally used to predict oral absorption, blood–brain barrier permeation, and P-glycoprotein-mediated transport [59].

### 4.12. In Silico Toxicity Prediction

The toxicity profiles of the phenolic compounds identified in the mycelial extracts were analyzed using the ProTox-III web server https://tox.charite.de/protox3/ (accessed on 21 April 2026) [61]. Canonical SMILES of individual phenolic compounds were submitted separately to ProTox-III. Oral toxicity, toxicity class prediction, and organ-specific endpoints were assessed, including hepatotoxicity, nephrotoxicity, neurotoxicity, cardiotoxicity, and respiratory toxicity. Endpoints associated with carcinogenicity, immunotoxicity, mutagenicity, and cytotoxicity were also considered.

### 4.13. Statistical Analysis

Twenty independent biological replicates were produced for each cultivation system. Analytical determinations were performed in triplicate unless otherwise stated. Data obtained for phenolic compounds, antioxidant activity, photoprotective properties, and anti-tyrosinase activity were subjected to analysis of variance (ANOVA), and means were compared using the Scott–Knott test at a 5% significance level (*p* ≤ 0.05). Statistical analyses were performed using Sisvar version 5.8 [75].

## 5. Conclusions

This study demonstrated that axenic and paired cultivation of basidiomycetes influences both the phenolic composition and the biological properties of mycelial extracts. Co-cultivation modified the phenolic profiles of the fungi and promoted the occurrence of compounds not detected under axenic conditions, indicating that interactions among the four basidiomycetes can influence secondary metabolite production. The extracts exhibited antioxidant activity, broad-spectrum photoprotection, and strong tyrosinase inhibition, highlighting their potential as sources of bioactive compounds of cosmetic interest. Computational analyses provided complementary information and suggested associations between the identified phenolics and biological processes associated with oxidative stress responses, UV adaptation, and melanin regulation. Together, these results show that fungal co-cultivation can alter the phenolic composition of basidiomycete mycelia and indicate that co-cultivation of basidiomycetes may be a useful strategy for obtaining extracts enriched in bioactive metabolites with potential relevance to cosmetic and biotechnological applications.

## Figures and Tables

**Table 1:** Phenolic compounds (µg g^−1^ DW) of the mycelial biomass from basidiomycetes cultivated alone or in paired cultures.

	Benzoic Acid (243) *	Vanillin (1183) *	Protocatechuic Acid (72) *	Gallic Acid (370) *	Syringic Acid (10,742) *	*p*-Coumaric Acid (637,542) *	Catechin (9064) *	Quercetin (5,280,343) *	Myricetin (5,281,672) *	Total
LC	4.7 ± 0.1 ^eD^	4.4 ± 0.2 ^fC^	5.7 ± 0.1 ^cE^	13.50± 0.0 ^bE^	nd	5.2 ± 0.6 ^dB^	nd	16.9 ± 0.6 ^aE^	3.1 ± 0.2 ^gA^	53.5 ± 0.2
PO	60.5 ± 0.2 ^aA^	4.7 ± 0.0 ^dA^	5.9 ± 0.1 ^cE^	26.3 ± 0.4 ^bB^	nd	nd	nd	nd	nd	97.4 ± 0.6
PS	nd	tr	41.8 ± 0.1 ^bA^	17.8 ± 0.5 ^cC^	nd	nd	nd	52.1 ± 0.9 ^aA^	nd	111.7 ± 1.7
TP	5.7 ± 0.2 ^dC^	4.5 ± 0.0 ^eB^	37.6 ± 0.0 ^aB^	16.0 ± 0.6 ^bD^	nd	4.30 ± 0.0 ^eC^	nd	14.8 ± 0.0 ^cG^	nd	83.0 ± 0.3
LC + PS	4.2 ± 0.0 ^dE^	4.7 ± 0.0 ^dA^	nd	15.6 ± 0.4 ^bD^	nd	5.9 ± 0.1 ^cA^	nd	39.5 ± 0.0 ^aB^	nd	69.9 ± 0.6
LC + TP	nd	4.5 ± 0.0 ^dB^	nd	16.7 ± 0.5 ^aC^	nd	5.1 ± 0.0 ^dB^	11.9 ± 0.0 ^cB^	15.1 ± 0.0 ^bG^	nd	53.4 ± 0.5
LC + PO	5.0 ± 0.0 ^eD^	nd	7.3 ± 0.4 ^dD^	17.5 ± 0.6 ^aC^	4.9 ± 0.1 ^eA^	5.1 ± 0.0 ^eB^	13.2 ± 0.3 ^cA^	15.7 ± 0.0 ^bF^	nd	68.6 ± 1.2
PS + TP	3.8 ± 0.0 ^eE^	4.4 ± 0.0 ^eC^	25.0 ± 0.7 ^bC^	14.7 ± 0.1 ^cD^	nd	4.4 ± 0.2 ^eC^	7.0 ± 0.0 ^dC^	36.8 ± 0.1 ^aC^	nd	96.2 ± 0.9
PO + PS	8.5 ± 0.5 ^bB^	4.5 ± 0.0 ^cB^	nd	18.6 ± 0.6 ^aC^	4.4 ± 0.0 ^cA^	4.4 ± 0.1 ^cC^	nd	nd	nd	39.9 ± 2.1
PO + TP	nd	4.3 ± 0.0 ^dC^	8.0 ± 0.1 ^cD^	41.0 ± 1.4 ^aA^	nd	4.1 ± 0.0 ^dC^	nd	18.0 ± 0.0 ^bD^	nd	74.5 ± 3.0

* (PubChem ID). Values represent the mean ± standard deviation. According to the Scott-Knott test, means indicated by distinct letters show a statistical difference (*p* ≤ 0.05). Lowercase letters on the same row indicate significant differences in the concentrations of phenolic compounds. Uppercase letters in the same column indicate significant differences in mono and co-cultures. nd = not detected, tr = trace. LC = *Lentinus crinitus*, PO = *Pleurotus ostreatus*, PS = *Pycnoporus sanguineus*, and TP = *Trametes polyzona*.

**Table 2:** Total phenol content and antioxidant activity of the mycelial biomass of basidiomycetes cultivated alone or in paired cultures.

	Total Phenols (µg GAE mg^−1^)	DPPH• (IC_50_ in mg mL^−1^)	FRAP (µmol Fe^2+^ g^−1^)	BCLA (%)
*Lentinus crinitus*	31.96 ± 1.10 ^d^	130.40 ± 1.97 ^d^	9.47 ± 0.04 ^e^	57.00 ± 1.97 ^b^
*Pleurotus ostreatus*	32.01 ± 0.01 ^d^	133.00 ± 1.83 ^d^	7.85 ± 0.05 ^g^	56.00 ± 0.89 ^b^
*Pycnoporus sanguineus*	72.27 ± 0.83 ^b^	76.45 ± 0.45 ^b^	28.30 ± 0.46 ^b^	75.00 ± 3.33 ^a^
*Trametes polyzona*	78.21 ± 0.43 ^a^	64.40 ± 0.64 ^a^	34.63 ± 0.16 ^a^	51.05 ± 5.03 ^c^
*Lentinus + Pycnoporus*	29.75 ± 1.30 ^d^	147.00 ± 1.83 ^e^	10.53 ± 0.11 ^d^	35.77 ± 4.75 ^d^
*Lentinus + Trametes*	20.64 ± 0.65 ^e^	184.00 ± 4.83 ^f^	7.86 ± 0.13 ^g^	24.20 ± 2.42 ^e^
*Lentinus + Pleurotus*	21.15 ± 0.97 ^e^	217.00 ± 7.21 ^h^	6.50 ± 0.08 ^h^	21.00 ± 3.39 ^f^
*Pycnoporus + Trametes*	43.48 ± 1.03 ^c^	123.00 ± 1.32 ^c^	18.85 ± 0.77 ^c^	27.57 ± 2.80 ^e^
*Pleurotus + Pycnoporus*	21.72 ± 1.39 ^e^	236.00 ± 9.23 ^i^	7.00 ± 0.11 ^h^	39.00 ± 2.31 ^d^
*Pleurotus + Trametes*	29.81 ± 1.00 ^d^	194.20 ± 1.73 ^g^	8.50 ± 0.05 ^f^	40.54 ± 4.42 ^d^

Values represent the mean ± standard deviation (dry basis). Different letters in the same column indicate significant differences according to the Skott-Knott test (*p* ≤ 0.05; *n* = 3). IC_50_ = half-maximal inhibitory concentration. Positive controls: Quercetin IC_50_ = 0.02 ± 0,1 mg mL^−1^ (DPPH∙), Trolox IC_50_ = 10.5 ± 0.9 µmol Fe^+2^ g^−1^ (FRAP). Trolox *β*-carotene bleaching inhibition = 89.80 ± 4.13% (BCLA).

**Table 3:** Sun protection factor (SPF) of the mycelial biomass extract from basidiomycetes grown alone or in paired cultures.

	Extract Concentration (mg mL^−1^)				
	10	20	30	40	50
*Lentinus crinitus*	7.69 ± 0.24 ^eD^	15.44 ± 0.12 ^dF^	22.90 ± 0.30 ^cG^	30.24 ± 0.51 ^bE^	35.22 ± 0.15 ^aD^
*Pleurotus ostreatus*	15.00 ± 0.45 ^dA^	30.83 ± 0.14 ^cA^	38.70 ± 0.10 ^bA^	39.33 ± 0.00 ^aA^	39.33 ± 0.00 ^aA^
*Pycnoporus sanguineus*	7.18 ± 0.08 ^eE^	14.55 ± 0.44 ^dG^	22.62 ± 0.51 ^cG^	29.17 ± 0.16 ^bF^	33.69 ± 0.28 ^aF^
*Trametes polyzona*	14.69 ± 0.53 ^dA^	29.61 ± 0.68 ^cB^	37.46 ± 0.19 ^bB^	39.13 ± 0.12 ^aA^	39.33 ± 0.00 ^aA^
*Lentinus + Pycnoporus*	9.52 ± 0.20 ^eC^	20.25 ± 0.48 ^dD^	30.33 ± 0.36 ^cD^	38.02 ± 0.08 ^bB^	39.30 ± 0.01 ^aA^
*Lentinus + Trametes*	5.32 ± 0.07 ^eF^	11.93 ± 0.16 ^dH^	17.69 ± 0.43 ^cI^	23.37 ± 0.31 ^bH^	28.83 ± 0.37 ^aG^
*Lentinus + Pleurotus*	7.04 ± 0.35 ^eE^	14.54 ± 0.18 ^dG^	22.05 ± 0.17 ^cH^	28.56 ± 0.43 ^bG^	34.26 ± 0.09 ^aE^
*Pycnoporus + Trametes*	11.74 ± 0.66 ^dB^	24.08 ± 0.27 ^cC^	34.72 ± 0.80 ^bC^	39.03 ± 0.08 ^aA^	39.33 ± 0.00 ^aA^
*Pleurotus + Pycnoporus*	7.57 ± 0.31 ^eD^	15.83 ± 0.09 ^dF^	24.04 ± 0.04 ^cF^	32.15 ± 0.59 ^bD^	37.34 ± 0.22 ^aB^
*Pleurotus + Trametes*	7.97 ± 0.36 ^eD^	16.38 ± 0.25 ^dE^	25.37 ± 0.31 ^cE^	33.10 ± 0.31 ^bC^	36.50 ± 0.95 ^aC^

Values represent the mean ± standard deviation. According to the Scott-Knott test, means indicated by distinct letters show a statistical difference (*p* ≤ 0.05). Lowercase letters on the same row indicate significant differences in the SPF of distinct extract concentrations. Uppercase letters in the same column indicate significant differences in mono and co-cultures.

**Table 4:** UVA/UVB ratio and UVA protection of the mycelial biomass extract from basidiomycetes grown alone or in paired cultures.

	UVA/UVB Ratio and Anti-UVA Protection				
	10 mg mL^−1^	20 mg mL^−1^	30 mg mL^−1^	40 mg mL^−1^	50 mg mL^−1^
*Lentinus crinitus*	0.89 ± 0.01 ^eD^ Superior	0.91 ± 0.00 ^dD^ Ultra	0.93 ± 0.01 ^cC^ Ultra	0.95 ± 0.00 ^bE^ Ultra	1.05 ± 0.00 ^aD^ Ultra
*Pleurotus ostreatus*	0.94 ± 0.01 ^eC^ Ultra	0.98 ± 0.00 ^dC^ Ultra	1.19 ± 0.01 ^cA^ Ultra	1.54 ± 0.02 ^bA^ Ultra	1.84 ± 0.02 ^aA^ Ultra
*Pycnoporus sanguineus*	0.75 ± 0.01 ^eF^ Good	0.77 ± 0.01 ^dF^ Good	0.79 ± 0.01 ^cG^ Good	0.81 ± 0.00 ^bG^ Superior	0.87 ± 0.02 ^aH^ Superior
*Trametes polyzona*	0.67 ± 0.00 ^eI^ Good	0.71 ± 0.00 ^dI^ Good	0.84 ± 0.01 ^cF^ Superior	1.02 ± 0.03 ^bD^ Ultra	1.28 ± 0.01 ^aC^ Ultra
*Lentinus + Pycnoporus*	1.17 ± 0.00 ^dA^ Ultra	1.19 ± 0.00 ^cA^ Ultra	1.19 ± 0.01 ^cA^ Ultra	1.33 ± 0.00 ^bB^ Ultra	1.59 ± 0.01 ^aB^ Ultra
*Lentinus + Trametes*	0.85 ± 0.00 ^eE^ Superior	0.86 ± 0.00 ^dE^ Superior	0.88 ± 0.00 ^cH^ Superior	0.89 ± 0.00 ^bF^ Superior	0.93 ± 0.01 ^aF^ Ultra
*Lentinus + Pleurotus*	0.71 ± 0.01 ^eH^ Good	0.73 ± 0.00 ^dH^ Good	0.75 ± 0.00 ^cG^ Good	0.80 ± 0.00 ^bG^ Superior	0.84 ± 0.00 ^aI^ Superior
*Pycnoporus + Trametes*	0.95 ± 0.01 ^eB^ Ultra	0.99 ± 0.00 ^dB^ Ultra	1.08 ± 0.01 ^cB^ Ultra	1.26 ± 0.02 ^bC^ Ultra	1.57 ± 0.01 ^aB^ Ultra
*Pleurotus + Pycnoporus*	0.85 ± 0.00 ^dE^ Superior	0.86 ± 0.00 ^cE^ Superior	0.86 ± 0.01 ^cE^ Superior	0.90 ± 0.00 ^bF^ Ultra	1.00 ± 0.01 ^aE^ Ultra
*Pleurotus + Trametes*	0.72 ± 0.00 ^dG^ Good	0.73 ± 0.00 ^cG^ Good	0.73 ± 0.01 ^cI^ Good	0.78 ± 0.01 ^bH^ Good	0.90 ± 0.01 ^aG^ Ultra

Values represent the mean ± standard deviation. According to the Scott-Knott test, means indicated by distinct letters show a statistical difference (*p* ≤ 0.05). Lowercase letters on the same row indicate significant differences in the UVA/UVB ratio of distinct extract concentrations. Uppercase letters in the same column indicate significant differences in mono and co-cultures. Classification of UVA protection according to Boots’ star rating [32].

**Table 5:** Selected enriched GO biological processes for axenic cultures.

Extract	GO Term	GO ID	FDR	Functional Category
*L. crinitus*	response to oxidative stress	GO:0006979	4.00 × 10^−7^	Antioxidant
*L. crinitus*	cellular response to oxidative stress	GO:0034599	1.63 × 10^−6^	Antioxidant
*L. crinitus*	cellular response to UV-A	GO:0071492	3.34 × 10^−5^	UV response
*L. crinitus*	inflammatory response	GO:0006954	3.92 × 10^−6^	Inflammation
*L. crinitus*	regulation of inflammatory response	GO:0050727	1.58 × 10^−6^	Inflammation
*L. crinitus*	regulation of apoptotic process	GO:0042981	4.33 × 10^−3^	Apoptosis
*L. crinitus*	response to stress	GO:0006950	4.00 × 10^−7^	Cellular stress
*P. sanguineus*	response to oxidative stress	GO:0006979	1.20 × 10^−6^	Antioxidant
*P. sanguineus*	cellular response to oxidative stress	GO:0034599	3.40 × 10^−6^	Antioxidant
*P. sanguineus*	cellular response to UV-A	GO:0071492	2.10 × 10^−5^	UV response
*P. sanguineus*	inflammatory response	GO:0006954	4.80 × 10^−6^	Inflammation
*P. sanguineus*	regulation of inflammatory response	GO:0050727	1.90 × 10^−6^	Inflammation
*P. sanguineus*	regulation of apoptotic process	GO:0042981	2.80 × 10^−4^	Apoptosis
*P. sanguineus*	response to stress	GO:0006950	1.60 × 10^−5^	Cellular stress
*T. polyzona*	response to oxidative stress	GO:0006979	8.70 × 10^−7^	Antioxidant
*T. polyzona*	cellular response to oxidative stress	GO:0034599	2.10 × 10^−6^	Antioxidant
*T. polyzona*	cellular response to UV-A	GO:0071492	1.80 × 10^−5^	UV response
*T. polyzona*	inflammatory response	GO:0006954	5.10 × 10^−6^	Inflammation
*T. polyzona*	regulation of inflammatory response	GO:0050727	2.40 × 10^−6^	Inflammation
*T. polyzona*	regulation of apoptotic process	GO:0042981	3.10 × 10^−4^	Apoptosis
*T. polyzona*	response to stress	GO:0006950	1.70 × 10^−5^	Cellular stress

**Table 6:** Selected enriched GO biological processes for paired cultures.

Extract	GO Term	GO ID	FDR	Functional Category
*L. crinitus + P. ostreatus*	response to oxidative stress	GO:0006979	3.80 × 10^−7^	Antioxidant
*L. crinitus + P. ostreatus*	cellular response to UV-A	GO:0071492	1.40 × 10^−5^	UV response
*L. crinitus + P. ostreatus*	inflammatory response	GO:0006954	2.90 × 10^−6^	Inflammation
*L. crinitus + P. ostreatus*	regulation of apoptotic process	GO:0042981	1.90 × 10^−4^	Apoptosis
*L. crinitus + P. sanguineus*	response to oxidative stress	GO:0006979	2.90 × 10^−7^	Antioxidant
*L. crinitus + P. sanguineus*	cellular response to UV-A	GO:0071492	1.10 × 10^−5^	UV response
*L. crinitus + P. sanguineus*	inflammatory response	GO:0006954	2.20 × 10^−6^	Inflammation
*L. crinitus + P. sanguineus*	regulation of apoptotic process	GO:0042981	1.50 × 10^−4^	Apoptosis
*L. crinitus + T. polyzona*	response to oxidative stress	GO:0006979	2.60 × 10^−7^	Antioxidant
*L. crinitus + T. polyzona*	cellular response to UV-A	GO:0071492	9.70 × 10^−6^	UV response
*L. crinitus + T. polyzona*	inflammatory response	GO:0006954	2.00 × 10^−6^	Inflammation
*L. crinitus + T. polyzona*	regulation of apoptotic process	GO:0042981	1.30 × 10^−4^	Apoptosis
*P. ostreatus + P. sanguineus*	cellular response to UV-A	GO:0071492	1.44 × 10^−3^	UV response
*P. ostreatus + P. sanguineus*	regulation of inflammatory response	GO:0050727	3.47 × 10^−2^	Inflammation
*P. ostreatus + P. sanguineus*	regulation of apoptotic process	GO:0042981	5.34 × 10^−5^	Apoptosis
*P. ostreatus + T. polyzona*	response to oxidative stress	GO:0006979	2.80 × 10^−7^	Antioxidant
*P. ostreatus + T. polyzona*	cellular response to UV-A	GO:0071492	1.00 × 10^−5^	UV response
*P. ostreatus + T. polyzona*	inflammatory response	GO:0006954	2.30 × 10^−6^	Inflammation
*P. ostreatus + T. polyzona*	regulation of apoptotic process	GO:0042981	1.60 × 10^−4^	Apoptosis
*P. sanguineus + T. polyzona*	response to oxidative stress	GO:0006979	2.70 × 10^−7^	Antioxidant
*P. sanguineus + T. polyzona*	cellular response to UV-A	GO:0071492	9.20 × 10^−6^	UV response
*P. sanguineus + T. polyzona*	inflammatory response	GO:0006954	2.10 × 10^−6^	Inflammation
*P. sanguineus + T. polyzona*	regulation of apoptotic process	GO:0042981	1.40 × 10^−4^	Apoptosis

**Table 7:** PASS-predicted biological activities of phenolic compounds identified in basidiomycete mycelial extracts.

Compounds	Type of Predicted Activity (Pa > Pi)				
	Antioxidant	Free Radical Scavenger	Lipid Peroxidase Inhibitor	NADPH Oxidase Inhibitor	Melanin Inhibitor
Benzoic acid	–	0.372 > 0.020	0.370 > 0.044	0.355 > 0.007	–
Vanillin	0.403 > 0.012	0.546 > 0.008	0.711 > 0.005	0.305 > 0.011	–
Protocatechuic acid	0.401 > 0.012	0.420 > 0.030	0.516 > 0.015	0.488 > 0.004	0.310 > 0.011
Gallic acid	0.520 > 0.006	0.570 > 0.007	0.554 > 0.012	0.509 > 0.004	–
Syringic acid	0.403 > 0.012	0.619 > 0.005	0.559 > 0.012	0.520 > 0.004	–
*p*-Coumaric acid	0.553 > 0.005	0.627 > 0.005	0.529 > 0.014	–	0.433 > 0.004
Catechin	0.810 > 0.003	0.842 > 0.002	0.888 > 0.003	–	0.548 > 0.044
Quercetin	0.872 > 0.003	0.811 > 0.003	0.788 > 0.004	0.928 > 0.002	0.538 > 0.003
Myricetin	0.924 > 0.003	0.832 > 0.002	0.836 > 0.003	0.939 > 0.001	0.503 > 0.003

Pa = probability of activity; Pi = probability of inactivity. Only activities with Pa > Pi were considered. Activities are presented only when Pa ≥ 0.30. According to PASS recommendations, Pa > 0.70 indicates a high probability of experimental activity, 0.50 < Pa ≤ 0.70 indicates a moderate probability, and Pa ≤ 0.50 indicates a low probability of activity. The symbol (–) indicates that no relevant prediction was obtained according to the selected criteria.

**Table 8:** SwissADME-predicted physicochemical properties, pharmacokinetic profiles, and drug-likeness characteristics of phenolic compounds identified in basidiomycete mycelial biomass.

Parameters	Benzoic Acid	Vanillin	Protocatechuic Acid	Gallic Acid	Syringic Acid	*p*-Coumaric Acid	Catechin	Quercetin	Myricetin
Molecular weight (g/mol)	122.12	152.15	154.12	170.12	198.17	164.16	290.27	302.24	318.24
Fraction Csp^3^	0.00	0.12	0.00	0.00	0.22	0.00	0.20	0.00	0.00
Num. rotatable bonds	1	2	1	1	3	2	1	1	1
Num. H-bond acceptors	2	3	4	5	5	3	6	7	8
Num. H-bond donors	1	1	3	4	2	2	5	5	6
Molar Refractivity	33.40	40.34	37.45	39.47	48.41	45.13	74.33	78.03	80.06
TPSA	37.30 Å^2^	46.53 Å^2^	77.76 Å^2^	97.99 Å^2^	75.99 Å^2^	57.53 Å^2^	110.38 Å^2^	131.36 Å^2^	151.59 Å^2^
Log *P*_o/w_ (WLOGP)	1.38	1.21	0.80	0.50	1.11	1.38	1.22	1.99	1.69
Consensus Log *P*_o/w_	1.44	1.20	0.65	0.21	0.99	1.26	0.83	1.23	0.79
Log *S* (ESOL)	−2.20	−1.82	−1.86	−1.64	−1.84	−2.02	−2.22	−3.16	−3.01
Solubility mg/mL mol/L	7.66 × 10^−1^ 6.27 × 10^−3^	2.32 1.52 × 10^−2^	2.14 1.39 × 10^−2^	3.90 2.29 × 10^−2^	2.84 1.44 × 10^−2^	1.58 9.65 × 10^−3^	1.74 5.98 × 10^−3^	0.21 6.98 × 10^−4^	0.31 9.88 × 10^−4^
Qualitative water solubility	Soluble	Very soluble	Very soluble	Very soluble	Very soluble	Soluble	Soluble	Soluble	Soluble
GI absorption	High	High	High	High	High	High	High	High	Low
BBB permeant	Yes	Yes	No	No	No	Yes	No	No	No
P-gp substrate	No	No	No	No	No	No	Yes	No	No
CYP1A2 inhibitor	No	No	No	No	No	No	No	Yes	Yes
CYP2C9 inhibitor	No	No	No	No	No	No	No	No	No
CYP2C19 inhibitor	No	No	No	No	No	No	No	No	No
CYP2D6 inhibitor	No	No	No	No	No	No	No	Yes	No
CYP3A4 inhibitor	No	No	Yes	Yes	No	No	No	Yes	Yes
Log *K*_p_ (skin permeation)	−5.72 cm/s	−6.37 cm/s	−6.42 cm/s	−6.84 cm/s	−6.77 cm/s	−6.26 cm/s	−7.82 cm/s	−7.05 cm/s	−7.40 cm/s
Lipinski rule (Pfizer)	Yes 0 violation	Yes; 0 violation	Yes; 0 violation	Yes; 0 violation	Yes 0 violation	Yes 0 violation	Yes 0 violation	Yes 0 violation	Yes 1 violation: OH > 5
Ghose rule	No 3 violations: MW < 160 MR < 40 #atoms < 20	No 2 violations: MW < 16 #atoms < 20	No 3 violations: MW < 160 MR < 40 #atoms < 20	No 2 violations: MR < 40 #atoms < 20	Yes	Yes	Yes	Yes	Yes
Veber rule (GSK)	Yes	Yes	Yes	Yes	Yes	Yes	Yes	Yes	No 1 violation: TPSA > 140
Egan rule (Pharmacia)	Yes	Yes	Yes	Yes	Yes	Yes	Yes	Yes	No 1 violation: TPSA > 131.6
Muegge rule (Bayer)	No 1 violation: MW < 200	No 1 violation: MW < 200	No 1 violation: MW < 200	No 1 violation: MW < 200	No 1 violation: MW < 200	No 1 violation: MW < 200	Yes	Yes	No 2 violations: TPSA > 150; H-don > 5
Bioavailability Score	0.85	0.55	0.56	0.56	0.56	0.85	0.55	0.55	0.55

MW, molecular weight; TPSA, topological polar surface area; Log P, lipophilicity; Log S, aqueous solubility; GI, gastrointestinal; BBB, blood–brain barrier; P-gp, P-glycoprotein. Drug-likeness was evaluated using the Lipinski, Ghose, Veber, Egan, and Muegge filters implemented in SwissADME.

**Table 9:** ProTox-III-predicted oral toxicity, organ toxicity, and toxicological endpoints of phenolic compounds identified in basidiomycete mycelial extracts.

Parameter	Benzoic Acid	Vanillin	Protocatechuic Acid	Gallic Acid	Syringic Acid	*p*-Coumaric Acid	Catechin	Quercetin	Myricetin
Oral toxicity prediction									
Predicted LD_50_ (mg/kg)	290	1000	2000	2000	1700	2850	10,000	159	159
Predicted toxicity class	III	IV	IV	IV	IV	V	VI	III	III
Organ toxicity (% probability)									
Hepatotoxicity	Active (54)	Inactive (52)	Inactive (59)	Inactive (61)	Inactive (58)	Inactive (51)	Inactive (72)	Inactive (69)	Inactive (69)
Neurotoxicity	Inactive (73)	Inactive (59)	Inactive (86)	Inactive (88)	Inactive (76)	Inactive (71)	Inactive (90)	Inactive (89)	Inactive (89)
Nephrotoxicity	Active (56)	Active (53)	Active (61)	Active (69)	Active (66)	Active (66)	Active (62)	Active (62)	Active (62)
Cardiotoxicity	Inactive (52)	Inactive (79)	Inactive (90)	Inactive (89)	Inactive (91)	Inactive (98)	Inactive (99)	Inactive (99)	Inactive (99)
Respiratory toxicity	Inactive (70)	Inactive (98)	Inactive (58)	Active (52)	Inactive (77)	Inactive (62)	Active (79)	Active (83)	Active (83)
Toxicity endpoint (% probability)									
Carcinogenicity	Inactive (74)	Inactive (60)	Active (72)	Active (56)	Inactive (70)	Inactive (50)	Inactive (51)	Active (68)	Active (68)
Immunotoxicity	Inactive (99)	Inactive (55)	Inactive (99)	Inactive (99)	Inactive (97)	Inactive (91)	Inactive (96)	Inactive (87)	Inactive (86)
Mutagenicity	Inactive (99)	Inactive (98)	Inactive (97)	Inactive (94)	Inactive (93)	Inactive (93)	Inactive (55)	Active (51)	Active (51)
Cytotoxicity	Inactive (86)	Inactive (83)	Inactive (90)	Inactive (91)	Inactive (97)	Inactive (81)	Inactive (84)	Inactive (99)	Inactive (99)

LD_50_, median lethal dose. Toxicity classes were assigned according to ProTox-III: Class I, fatal if swallowed (LD_50_ ≤ 5); Class II, fatal if swallowed (5 < LD_50_ ≤ 50); Class III, toxic if swallowed (50 < LD_50_ ≤ 300); Class IV, harmful if swallowed (300 < LD_50_ ≤ 2000); Class V, may be harmful if swallowed (2000 < LD_50_ ≤ 5000); and Class VI, non-toxic (LD_50_ > 5000). Active indicates a predicted toxicological effect, and inactive indicates the absence of the predicted effect.

## Data Availability

The datasets generated during and/or analyzed during the current study are available from the corresponding author upon reasonable request.

## Supplementary Materials

The following supporting information can be downloaded at: https://www.mdpi.com/article/10.3390/molecules31152569/s1, Table S1: Selected enriched KEGG pathways for axenic and paired cultures; Table S2: Dry biomass production of axenic and paired cultures of basidiomycetes after 22 days of cultivation; Table S3: Calibration curves and validation parameters (R^2^, LOD, and LOQ) of the phenolic standards used for HPLC quantification of compounds identified in basidiomycete mycelial extracts; Figure S1: HPLC chromatogram of the phenolic standards used for compound identification and quantification. Peaks: (1) gallic acid, (2) protocatechuic acid, (3) catechin, (4) syringic acid, (5) vanillin, (6) *p*-coumaric acid, (7) benzoic acid, (8) myricetin, and (9) quercetin; Figure S2: Representative HPLC chromatogram of phenolic compounds identified in the mycelial extract of *Lentinus crinitus*; Figure S3: Representative HPLC chromatogram of phenolic compounds identified in the mycelial extract of *Pleurotus ostreatus*; Figure S4: Representative HPLC chromatogram of phenolic compounds identified in the mycelial extract of *Pycnoporus sanguineus*; Figure S5: Representative HPLC chromatogram of phenolic compounds identified in the mycelial extract of *Trametes polyzona*; Figure S6: Representative HPLC chromatogram of phenolic compounds identified in the mycelial extract of the *Lentinus crinitus* and *Pycnoporus sanguineus* co-culture; Figure S7: Representative HPLC chromatogram of phenolic compounds identified in the mycelial extract of the *Lentinus crinitus* and *Trametes polyzona* co-culture; Figure S8: Representative HPLC chromatogram of phenolic compounds identified in the mycelial extract of the *Lentinus crinitus* and *Pleurotus ostreatus* co-culture; Figure S9: Representative HPLC chromatogram of phenolic compounds identified in the mycelial extract of the *Pycnoporus sanguineus* and *Trametes polyzona* co-culture; Figure S10: Representative HPLC chromatogram of phenolic compounds identified in the mycelial extract of the *Pleurotus ostreatus* and *Pycnoporus sanguineus* co-culture; Figure S11: Representative HPLC chromatogram of phenolic compounds identified in the mycelial extract of the *Pleurotus ostreatus* and *Trametes polyzona* co-culture.
