# Supplementary material for: Effects of Co-Cultivation on the Phenolic Composition and Bioactive Properties of Basidiomycete Mycelia: Antioxidant, Photoprotective, Anti-Tyrosinase, and In Silico Evidence
Source: Molecules. 2026 Jul 23;31(15):2569. doi: 10.3390/molecules31152569 (PMC13467801; doi:10.3390/molecules31152569)
Supplement: Supplementary file 1 [file molecules-31-02569-s001.zip › molecules-4399026-supplementary.pdf]

molecules

Supplementary Materials: Effects of Co-Cultivation on the Phenolic Composition and Bioactive Properties of Basidiomycete Mycelia: Antioxidant, Photoprotective, Anti-Tyrosinase, and In Silico Evidence

Katielle Vieira Avelino [1], Maria Aparecida Pereira Garcez [1], Roberta Fernanda Rogonni Ferrari Giansante [2], Marisangela Isabel Wietzikoski Halabura [1], Caroline Domingues [3], Maria Graciela Iecher Faria Nunes [1], Nelson Barros Colauto [4], Lidiane Nunes Barbosa [2,3], Zilda Cristiani Gazim [1,2], Daniela Dib Gonçalves [2,3], Flávio Augusto Vicente Seixas [5], Antonio Laverde Junior [6] and Juliana Silveira do Valle [1,2]*

[1] Graduate Program in Biotechnology Applied to Agriculture, Universidade Paranaense, Umuarama 87502-210, Brazil;
[2] Graduate Program in Animal Science with Emphasis on Bioactive Products, Universidade Paranaense, Umuarama 87502-210, Brazil;
[3] Graduate Program in Medicinal and Phytotherapeutic Plants in Primary Care, Universidade Paranaense, Umuarama 87502-210, Brazil;
[4] Universidade Tecnológica Federal do Paraná, Toledo, 85902-490, Brazil;
[5] Department of Technology, Universidade Estadual de Maringá, Umuarama, 87506-370, Brazil;
[6] Laboratório de Química Orgânica e Biomateriais, Departamento de Química, Universidade Tecnológica Federal do Paraná, Londrina, 86036-700, Brazil.

* Correspondence: jsvalle@prof.unipar.br

Table S1: Selected enriched KEGG pathways for axenic and paired cultures.

Extract	Pathway	KEGG ID	Functional category
L. crinitus	Chemical carcinogenesis – reactive oxygen species	hsa05208	Oxidative stress
L. crinitus	PI3K-Akt signaling pathway	hsa04151	Cell signaling/survival
L. crinitus	HIF-1 signaling pathway	hsa04066	Cellular stress response
L. crinitus	FoxO signaling pathway	hsa04068	Oxidative stress/apoptosis
L. crinitus	Lipid and atherosclerosis	hsa05417	Inflammation / oxidative stress
P. ostreatus	HIF-1 signaling pathway	hsa04066	Cellular stress response
P. ostreatus	Autophagy – animal	hsa04140	Cellular stress/survival
P. ostreatus	p53 signaling pathway	hsa04115	Apoptosis / cellular damage
P. ostreatus	Cysteine and methionine metabolism	hsa00270	Oxidative stress/redox
P. sanguineus	Chemical carcinogenesis – reactive oxygen species	hsa05208	Oxidative stress
P. sanguineus	PI3K-Akt signaling pathway	hsa04151	Cell signaling/survival
P. sanguineus	HIF-1 signaling pathway	hsa04066	Cellular stress response
P. sanguineus	FoxO signaling pathway	hsa04068	Oxidative stress/apoptosis
P. sanguineus	p53 signaling pathway	hsa04115	Apoptosis / cellular damage

Extract	Pathway	KEGG ID	Functional category
T. polyzona	Chemical carcinogenesis – reactive oxygen species	hsa05208	Oxidative stress
T. polyzona	PI3K-Akt signaling pathway	hsa04151	Cell signaling/survival
T. polyzona	HIF-1 signaling pathway	hsa04066	Cellular stress response
T. polyzona	FoxO signaling pathway	hsa04068	Oxidative stress/apoptosis
T. polyzona	p53 signaling pathway	hsa04115	Apoptosis / cellular damage
L. crinitus + *P. ostreatus*	PI3K-Akt signaling pathway	hsa04151	Cell signaling/survival
L. crinitus + *P. ostreatus*	Chemical carcinogenesis – reactive oxygen species	hsa05208	Oxidative stress
L. crinitus + *P. ostreatus*	HIF-1 signaling pathway	hsa04066	Cellular stress response
L. crinitus + *P. ostreatus*	FoxO signaling pathway	hsa04068	Oxidative stress/apoptosis
L. crinitus + *P. ostreatus*	p53 signaling pathway	hsa04115	Apoptosis / cellular damage
L. crinitus + *P. sanguineus*	PI3K-Akt signaling pathway	hsa04151	Cell signaling/survival
L. crinitus + *P. sanguineus*	Chemical carcinogenesis – reactive oxygen species	hsa05208	Oxidative stress
L. crinitus + *P. sanguineus*	HIF-1 signaling pathway	hsa04066	Cellular stress response
L. crinitus + *P. sanguineus*	FoxO signaling pathway	hsa04068	Oxidative stress/apoptosis
L. crinitus + *P. sanguineus*	p53 signaling pathway	hsa04115	Apoptosis / cellular damage
L. crinitus + *T. polyzona*	PI3K-Akt signaling pathway	hsa04151	Cell signaling/survival
L. crinitus + *T. polyzona*	Chemical carcinogenesis – reactive oxygen species	hsa05208	Oxidative stress
L. crinitus + *T. polyzona*	HIF-1 signaling pathway	hsa04066	Cellular stress response
L. crinitus + *T. polyzona*	FoxO signaling pathway	hsa04068	Oxidative stress/apoptosis
L. crinitus + *T. polyzona*	p53 signaling pathway	hsa04115	Apoptosis / cellular damage
P. ostreatus + *P. sanguineus*	HIF-1 signaling pathway	hsa04066	Cellular stress response
P. ostreatus + *P. sanguineus*	Lipid and atherosclerosis	hsa05417	Inflammation / oxidative stress
P. ostreatus + *P. sanguineus*	AGE-RAGE signaling pathway	hsa04933	Inflammation / oxidative stress
P. ostreatus + *P. sanguineus*	Arachidonic acid metabolism	hsa00590	Inflammation
P. ostreatus + *T. polyzona*	PI3K-Akt signaling pathway	hsa04151	Cell signaling/survival
P. ostreatus + *T. polyzona*	Chemical carcinogenesis – reactive oxygen species	hsa05208	Oxidative stress
P. ostreatus + *T. polyzona*	HIF-1 signaling pathway	hsa04066	Cellular stress response
P. ostreatus + *T. polyzona*	FoxO signaling pathway	hsa04068	Oxidative stress/apoptosis
P. ostreatus + *T. polyzona*	p53 signaling pathway	hsa04115	Apoptosis / cellular damage
P. sanguineus + *T. polyzona*	PI3K-Akt signaling pathway	hsa04151	Cell signaling/survival
P. sanguineus + *T. polyzona*	Chemical carcinogenesis – reactive oxygen species	hsa05208	Oxidative stress
P. sanguineus + *T. polyzona*	HIF-1 signaling pathway	hsa04066	Cellular stress response
P. sanguineus + *T. polyzona*	FoxO signaling pathway	hsa04068	Oxidative stress/apoptosis
P. sanguineus + *T. polyzona*	p53 signaling pathway	hsa04115	Apoptosis / cellular damage

Table S2. Dry biomass production of axenic and paired cultures of basidiomycetes after 22 days of cultivation.

Cultures	Mycelial biomass (mg/mL)
Lentinus crinitus	4,50
Pleurotus ostreatus	3,45
Pycnoporus sanguineus	6,23
Trametes polyzona	4,10
Lentinus+Pycnoporus	6,04
Lentinus+Trametes	3,72
Lentinus+Pleurotus	4,92
Pycnoporus+Trametes	5,00
Pleurotus+Pycnoporus	4,75
Pleurotus+Trametes	3,12

Table S3. Calibration curves and validation parameters (R^2, LOD, and LOQ) of the phenolic standards used for HPLC quantification of compounds identified in basidiomycete mycelial extracts.

Compound	Supplier	Linearity Range (μg/mL)	Calibration Equation	R^2	LOD (μg/mL)	LOQ (μg/mL)
Benzoic acid	Sigma-Aldrich	0 – 20	y = 7842,4x - 1151,7	0,9964	0,96	2,92
Catechin			y = 9162,9x - 1829,5	0,9989	0,98	2,96
Gallic acid			y = 35738x - 2756	0,9973	1,49	4,52
Myricetin			y = 14324x - 1025	0,9934	3,27	10,27
p-Coumaric acid			y = 124688x - 19318	0,997	1,41	4,27
Protocatechuic acid			y = 32777x - 5421,9	0,996	1,39	4,21
Quercetin			y = 10937x - 6245,8	0,9915	1,92	5,83
Syringic acid			y = 63562x - 10503	0,996	1,71	5,18
Vanillin			y = 149904x - 25787	0,9962	1,06	3,21

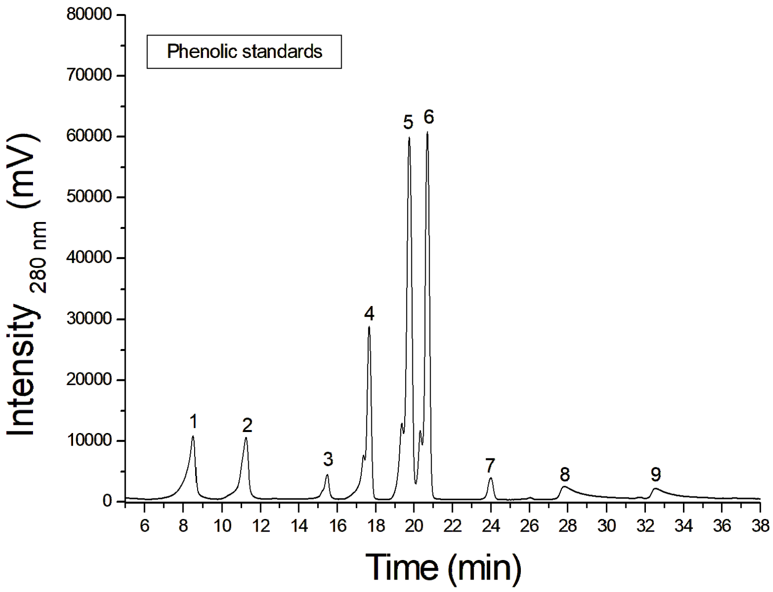

Figure S1. HPLC chromatogram of the phenolic standards used for compound identification and quantification. Peaks: (1) gallic acid, (2) protocatechuic acid, (3) catechin, (4) syringic acid, (5) vanillin, (6) *p*-coumaric acid, (7) benzoic acid, (8) myricetin, and (9) quercetin.

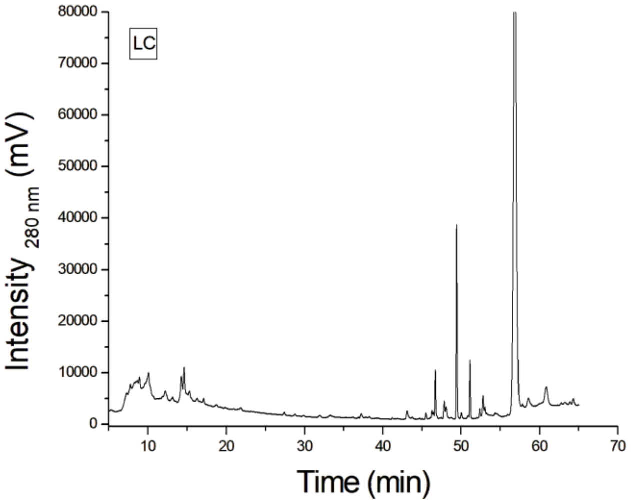

Figure S2. Representative HPLC chromatogram of phenolic compounds identified in the mycelial extract of *Lentinus crinitus*.

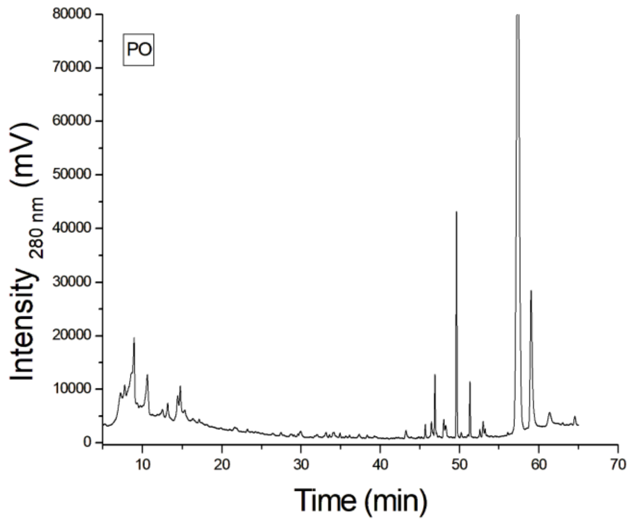

Figure S3. Representative HPLC chromatogram of phenolic compounds identified in the mycelial extract of *Pleurotus ostreatus*.

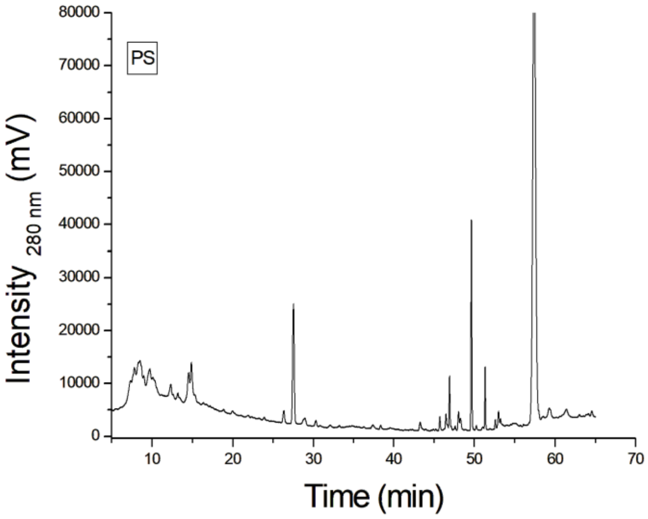

Figure S4. Representative HPLC chromatogram of phenolic compounds identified in the mycelial extract of *Pycnoporus sanguineus*.

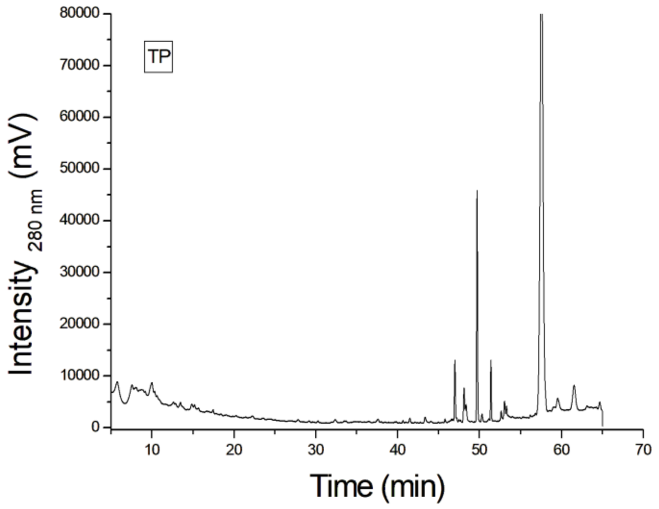

Figure S5. Representative HPLC chromatogram of phenolic compounds identified in the mycelial extract of *Trametes polyzona*.

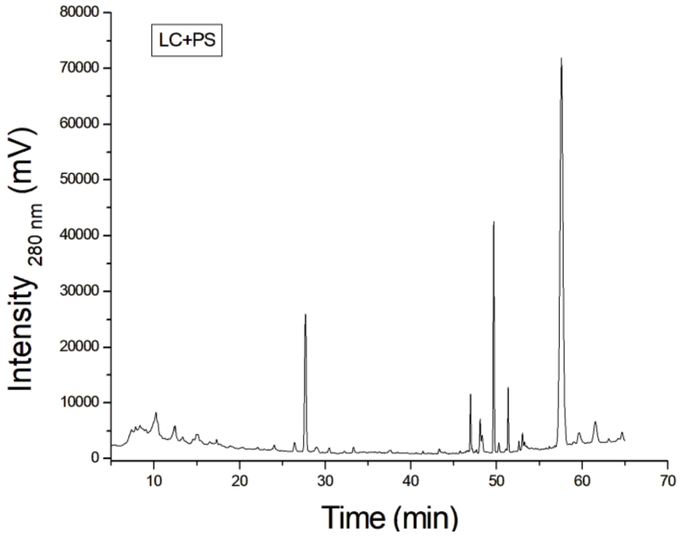

Figure S6. Representative HPLC chromatogram of phenolic compounds identified in the mycelial extract of the *Lentinus crinitus* and *Pycnoporus sanguineus* co-culture.

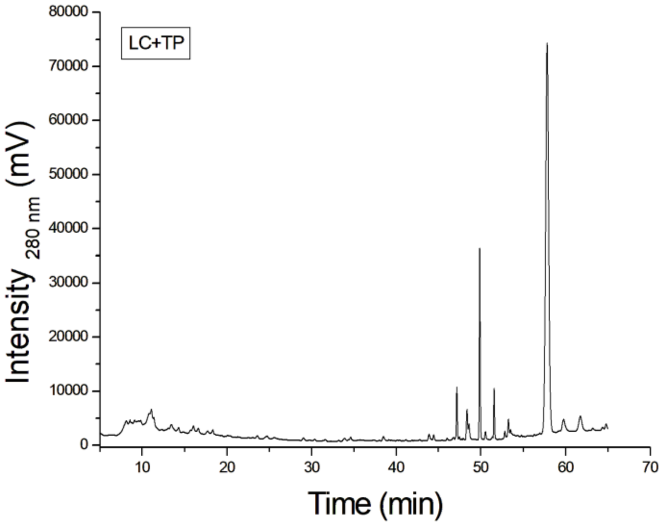

Figure S7. Representative HPLC chromatogram of phenolic compounds identified in the mycelial extract of the *Lentinus crinitus* and *Trametes polyzona* co-culture.

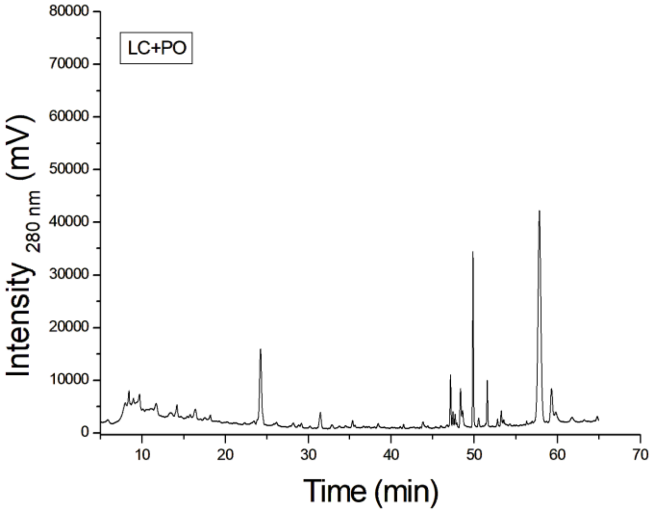

Figure S8. Representative HPLC chromatogram of phenolic compounds identified in the mycelial extract of the *Lentinus crinitus* and *Pleurotus ostreatus* co-culture.

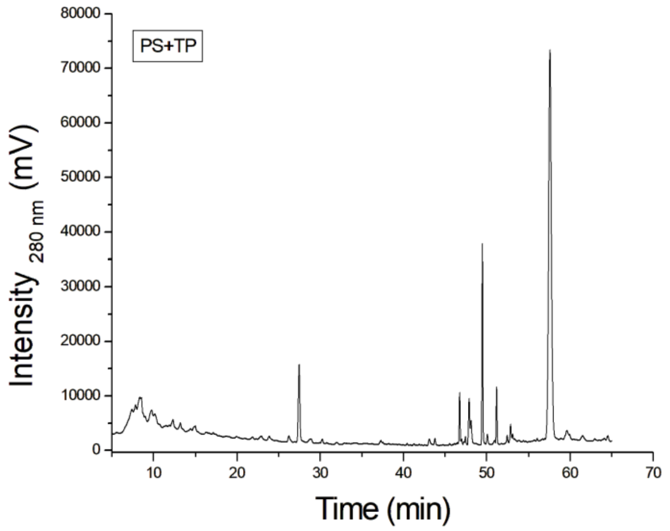

Figure S9. Representative HPLC chromatogram of phenolic compounds identified in the mycelial extract of the *Pycnoporus sanguineus* and *Trametes polyzona* co-culture.

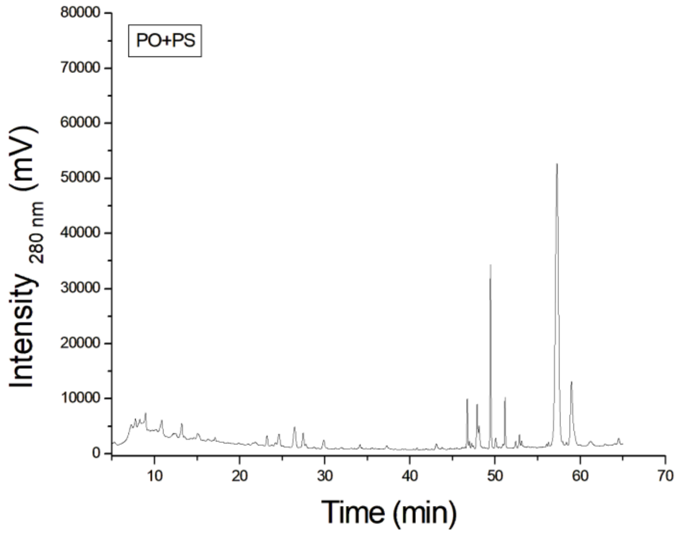

Figure S10. Representative HPLC chromatogram of phenolic compounds identified in the mycelial extract of the *Pleurotus ostreatus* and *Pycnoporus sanguineus* co-culture.

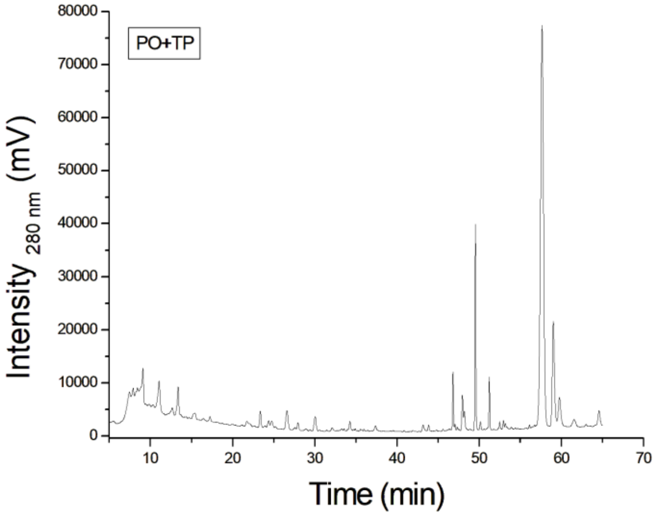

Figure S11. Representative HPLC chromatogram of phenolic compounds identified in the mycelial extract of the *Pleurotus ostreatus* and *Trametes polyzona* co-culture.
